# Nestin^+^ cells direct inflammatory cell migration in atherosclerosis

**DOI:** 10.1038/ncomms12706

**Published:** 2016-09-02

**Authors:** Raquel del Toro, Raphael Chèvre, Cristina Rodríguez, Antonio Ordóñez, José Martínez-González, Vicente Andrés, Simón Méndez-Ferrer

**Affiliations:** 1 Centro Nacional de Investigaciones Cardiovasculares Carlos III (CNIC), 28029 Madrid, Spain; 2 Grupo de Fisiopatología Cardiovascular, Instituto de Biomedicina de Sevilla (IBIS), 41013 Seville, Spain; 3 Centro de Investigación Cardiovascular (CSIC-ICCC), IIB-Sant Pau, 08025 Barcelona, Spain; 4 Wellcome Trust-Medical Research Council Cambridge Stem Cell Institute and Department of Haematology, University of Cambridge, and National Health Service Blood and Transplant, Cambridge Biomedical Campus, CB2 0PT Cambridge, UK

## Abstract

Atherosclerosis is a leading death cause. Endothelial and smooth muscle cells participate in atherogenesis, but it is unclear whether other mesenchymal cells contribute to this process. Bone marrow (BM) nestin^+^ cells cooperate with endothelial cells in directing monocyte egress to bloodstream in response to infections. However, it remains unknown whether nestin^+^ cells regulate inflammatory cells in chronic inflammatory diseases, such as atherosclerosis. Here, we show that nestin^+^ cells direct inflammatory cell migration during chronic inflammation. In Apolipoprotein E (*ApoE*) knockout mice fed with high-fat diet, BM nestin^+^ cells regulate the egress of inflammatory monocytes and neutrophils. In the aorta, nestin^+^ stromal cells increase ∼30 times and contribute to the atheroma plaque. *Mcp1* deletion in nestin^+^ cells—but not in endothelial cells only— increases circulating inflammatory cells, but decreases their aortic infiltration, delaying atheroma plaque formation and aortic valve calcification. Therefore, nestin expression marks cells that regulate inflammatory cell migration during atherosclerosis.

## Abstract (web summary)

Bone marrow cells producing the intermediate filament nestin guide monocyte egress to the bloodstream in response to infection. Here, the authors show that nestin-producing stromal cells direct inflammatory cell migration in atherosclerosis, and that stromal Mcp1 is crucial in this process.

## Graphical abstract

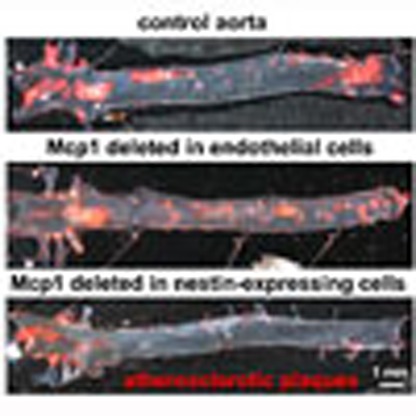

Atherosclerosis is a progressive chronic inflammatory disease characterized by the accumulation of leukocytes and cholesterol in the artery wall. It is initiated with the activation of endothelial cells, which recruit circulating inflammatory neutrophils and monocytes through adhesion molecules, like ICAM1 and VCAM1 (refs 1, 2), and increase the permeability for low-density lipoproteins (LDL) and other lipids, which also stimulate inflammatory infiltration^3^. Mice lacking Apolipoprotein E (ApoE) develop hypercholesterolaemia and atherosclerosis, more pronouncedly under high-fat diet (HFD)^4^,^5^. The chemokine monocyte chemotactic protein-1 (Mcp1) promotes leukocyte infiltration in the artery wall^6^. Infiltrated monocytes differentiate into macrophages, which engulf oxidized LDL and other lipids, becoming foam cells^7^. This process leads to the formation of the atheroma plaque, which enlarges as vascular cells proliferate and migrate from the media and the adventitia to the intima, where they produce the interstitial collagen and elastin that forms the fibrous cap. At advanced stages, this cap becomes fragile^8^ and sometimes calcified^9^. Plaque rupture induces blood coagulation, which can cause thrombosis, the major risk of atherosclerosis^10^.

Several studies have suggested that inflammatory cells can infiltrate the artery wall not only through the intima, but also through the adventitia^11^,^12^. Inflammatory infiltration is thus facilitated by the formation of a microvascular network called *vasa vasorum*, which provides oxygen and nutrients to the adventitia and the media^13^. In turn, inflammation can promote neovascularization, associated with bad prognosis and arterial occlusion^14^,^15^,^16^,^17^,^18^,^19^,^20^. Recent studies have suggested the presence of progenitors of endothelial and smooth muscle cells residing in the vascular wall^21^,^22^. Sca1^+^ cells isolated from the adventitia can differentiate into smooth muscle cells and endothelial cells, and contribute to atherosclerosis^23^. Conversely, another recent study showed that smooth muscle cells in the atheroma plaque can exhibit phenotypes of other cell lineages, including mesenchymal stem/progenitor cells (MSCs)^24^. Whereas stromal cells residing in the artery wall might exhibit certain degree of plasticity and contribute to atherogenesis, their crosstalk with inflammatory cells is only partially understood, and it remains unclear whether MSCs in the bone marrow (BM) and/or the aorta can directly regulate the traffic of inflammatory cells in chronic inflammatory diseases, such as atherosclerosis.

Monocytes and other inflammatory cells, such as neutrophils, are mainly produced in the BM. In response to pro-inflammatory cytokines or pathogens, monocytes egress from the BM, circulate and home to peripheral tissues, where they differentiate into macrophages and dendritic cells^25^. Only recently have the mechanisms that drive this directed migration began to be elucidated. One mechanism underlying the response to acute inflammation caused by infections involves MSCs expressing the intermediate filament protein nestin^26^, which cooperate with endothelial cells in directing the egress of inflammatory monocytes from the BM to circulation^27^. Mcp1 regulates the migration of these and other inflammatory cells through the CCR2 receptor expressed in monocytes, neutrophils and myeloid cells^28^. Some BM endothelial cells express nestin^29^,^30^, but it is unclear whether this expression is associated with specialized properties and functions.

In this study, we used a genetic approach to study side-by-side the role of endothelial cells and MSCs in the traffic of inflammatory cells and the formation of plaques in atherosclerosis. *Mcp1* deletion in nestin^+^ cells—but not in endothelial cells—increased BM egress of inflammatory cells, but reduced inflammatory infiltration in the aorta and significantly delayed atheroma plaque formation and aortic valve calcification. These results suggest that nestin^+^ stromal cells contribute to directed traffic of inflammatory cells in different tissues during chronic inflammation.

## Results

### BM nestin^+^ cells regulate inflammatory cell traffic

**Figure 1:**
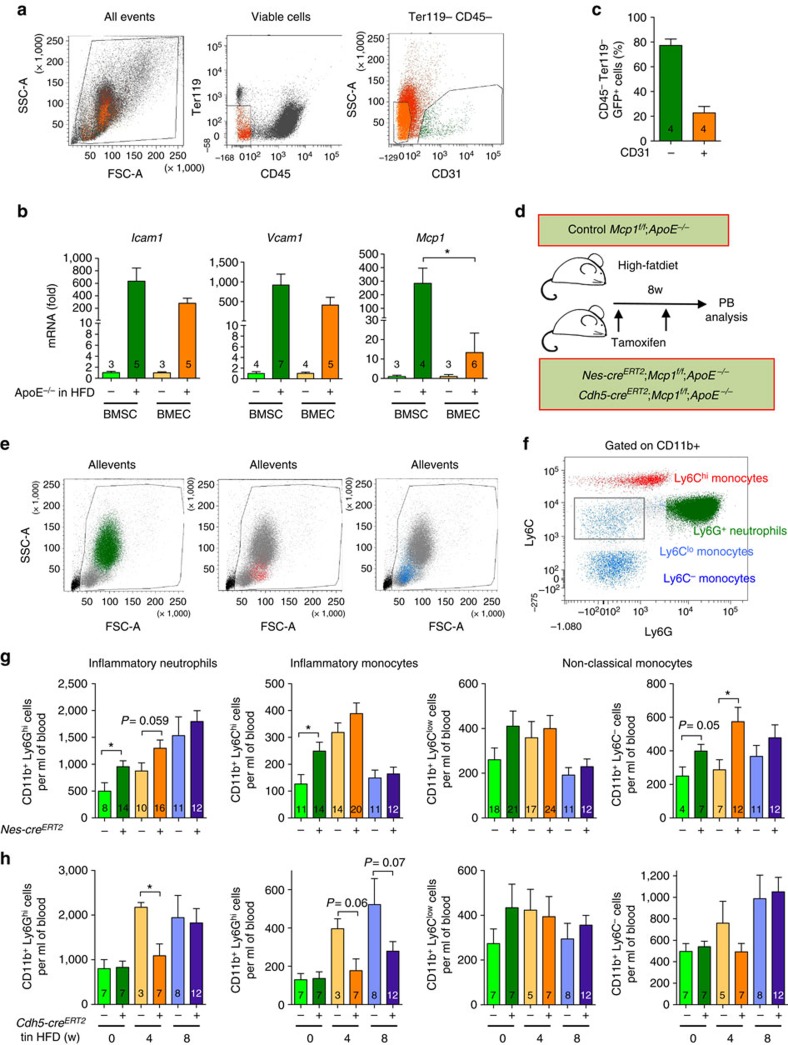
Nestin^+^ cells regulate inflammatory cell traffic in atherosclerosis. (**a**) Flow cytometry plots showing the cell sorting strategy. (**b**) QPCR analysis of *Icam1*, *Vcam1* and *Mcp1* mRNA in BM CD45^−^ Ter119^−^ CD31^+^ endothelial cells (BMEC) and BM CD45^−^ Ter119^−^ CD31^−^ stromal cells (BMSC) from *ApoE*^*−/−*^ mice fed with chow or HFD for 2 months (*n*=3–7). (**c**) CD31 expression in BM GFP^+^ stromal cells from *Nes-gfp;ApoE*^−/−^ mice fed with HFD for 2–3 months (*n*=4). (**d**) Experimental design of peripheral blood analysis from mice treated with tamoxifen and fed with HFD for 2 months. (**e**,**f**) Flow cytometry strategy used to identify inflammatory neutrophils, inflammatory monocytes and non-classical monocytes. (**g**,**h**) Number of non-classical monocytes, inflammatory monocytes and inflammatory neutrophils in the peripheral blood of tamoxifen-treated (**g**) *Nes-Cre*^*ERT2*^*;Mcp1*^*f/f*^;*ApoE*^−/−^ mice, (**h**) *Cdh5-Cre*^*ERT2*^*;Mcp1*^*f/f*^;*ApoE*^−/−^ mice and control littermates (*n*=3–12). Data are means±s.e.m.; *n* and *P* values are indicated; *P<0.05, unpaired two-tailed *t* test.

First, we studied the contribution of nestin^+^ stromal cells and endothelial cells to BM egress of inflammatory cells in atherosclerosis. We measured the expression of key adhesion molecules and chemokines in atherosclerosis in BM CD45^−^ Ter119^−^ CD31^−^ stromal cells (BMSCs) and CD45^−^ Ter119^−^ CD31^+^ endothelial cells (BMECs) isolated by fluorescence-activated cell sorting (FACS) from mice deficient in Apolipoprotein E (*ApoE*^*−/−*^) fed with chow diet or with HFD, to boost atherogenesis (Fig. 1a). The expression of the adhesion molecules Icam1 and Vcam1—required for trans-endothelial leukocyte migration^2^—was induced similarly in BMSCs and BMECs under HFD. In contrast, the induction of *Mcp1* expression was ∼30-fold higher in BMSCs (Fig. 1b), suggesting a possible role for BMSCs in regulating inflammatory monocyte migration in atherosclerosis.

To dissect the contribution of each cell population, we selectively deleted *Mcp1* in BMSCs and BMECs. Compared with other BM cells, BMSCs are enriched in the expression of the intermediate filament protein nestin^26^. We bred mice expressing the green fluorescent protein (GFP) under the regulatory elements of nestin promoter (*Nes-gfp*)^31^ with *ApoE*^*−/−*^ mice^4^. In compound mice, BM CD45^−^ Ter119^−^ GFP^+^ cells comprised ∼80% BMSCs and ∼20% BMECs (Fig. 1c). We deleted *Mcp1* in BMSCs using a transgenic mouse line expressing the inducible Cre^ERT2^ recombinase under the regulatory elements of the *Nestin* promoter (*Nes-cre*^*ERT2*^)^32^ bred to Mcp1-floxed mice^27^. For comparison, we also deleted Mcp1 specifically in endothelial cells using a line expressing Cre^ERT2^ under the regulatory elements of endothelial vascular cadherin (*Cdh5-cre*^ERT2^)^33^. Both Cre lines were intercrossed with ApoE-deficient mice to generate *Nes-cre*^*ERT2*^*;Mcp1*^*f/f*^*;ApoE*^*−/−*^ mice, *Cdh5-cre*^*ERT2*^*;Mcp1*^*f/f*^*;ApoE*^*−/−*^ mice and control *Mcp1*^*f/f*^*;ApoE*^*−/−*^ mice. We treated both groups of mice with tamoxifen (to induce Cre recombinase) and fed them with HFD for 8 weeks (Fig. 1d).

We measured circulating CD11b^+^ Ly6C^low^ and CD11b^+^ Ly6C^−^ non-classical monocytes, CD11b^+^ Ly6C^high^ inflammatory monocytes and CD11b^+^ Ly6G^high^ inflammatory neutrophils, soon after tamoxifen administration (3 days), or 4 and 8 weeks after feeding the mice with HFD (Fig. 1e–h). Mcp1 deletion in nestin^+^ cells rapidly and transiently increased the number of circulating inflammatory monocytes and neutrophils, whereas Mcp1 deletion in endothelial cells appeared to have the opposite effect (Fig. 1g,h). This was not explained by differential dyslipidemia, since the plasma concentration of cholesterol, low- and high-density lipoproteins remained unchanged (Supplementary Fig. 1a). These changes reflected an altered trafficking pattern of inflammatory cells, rather than proliferation of inflammatory cells in lymphoid organs; after 8–12 weeks of HFD, the number of inflammatory monocytes and neutrophils was unchanged in BM or spleen, which also showed a normal histological appearance (Supplementary Fig. 1b–e). Also, Mcp1 deletion in nestin^+^ cells did not alter the infiltration of inflammatory cells in other organs, such as the lung and the liver (Supplementary Fig. 1f,g). Similarly, *Cdh5-cre*^*ERT2*^*;Mcp1*^*f/f*^*;ApoE*^*−/−*^ mice also had normal numbers of splenocytes and BM nucleated cells, inflammatory monocytes and inflammatory neutrophils (Supplementary Fig. 2). The proliferation of macrophages locally in the atheroma plaque may represent an additional source of inflammatory cells in atherosclerosis^34^. We performed F4/80 staining in the aortic valves of *Nes-cre*^*ERT2*^*;Mcp1*^*f/f*^*;ApoE*^*−/−*^ mice and control *Mcp1*^*f/f*^*;ApoE*^*−/−*^ mice fed with HFD for 2 months and we did not observe any difference in the number of F4/80^+^ cells or in the number of Ki67^+^ cells (Supplementary Fig. 3). Altogether, these results suggest that nestin^+^ BMSCs regulate the egress of inflammatory cells from the BM to circulation during early stages of atherogenesis.

### Mcp1 deletion in nestin^+^ cells delays atherosclerosis

The egress of inflammatory cells from the BM towards circulation represents a first step in atherosclerosis as the main source of inflammatory cells that later infiltrate the artery wall. To determine the effect of Mcp1 deletion in BMSC or BMEC on atherosclerotic plaque formation, *Nes-cre*^*ERT2*^*;Mcp1*^*f/f*^*;ApoE*^*−/−*^ mice, *Cdh5-cre*^*ERT2*^*;Mcp1*^*f/f*^*;ApoE*^*−/−*^ mice and control *Mcp1*^*f/f*^*;ApoE*^*−/−*^ mice were fed with HFD for 2 months. After this period, their aortas were stained with Oil Red O to mark lipid accumulation in the artery wall. Planimetric measurements of atherosclerotic lesions were conducted in two regions: the thoracic aorta and the aortic arch, which contains the three main bifurcations exhibiting turbulent blood flow, resulting in more abundant and larger atherosclerotic plaques.

**Figure 2:**
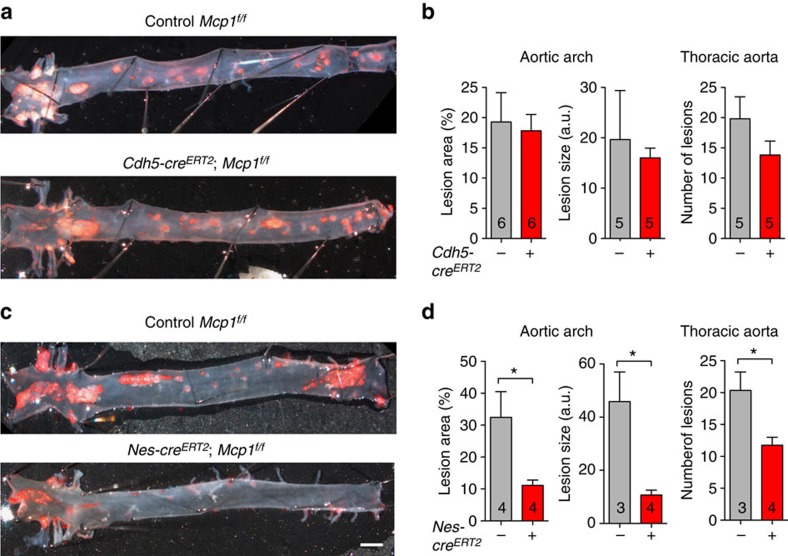
Mcp1 deletion in nestin^+^ cells delays atherosclerosis progression. (**a**,**c**) Representative photographs of whole-mounted aortas from tamoxifen-treated (**a**) *Cdh5-cre*^*ERT2*^*;Mcp1*^*f/f*^;*ApoE*^−/−^ mice, (**c**) *Nes-cre*^*ERT2*^*;Mcp1*^*f/f*^;*ApoE*^−/−^ mice and control littermates (without Cre) two months after HFD, stained with Oil Red O (red) to mark atheroma plaques (*n*=6). (**a**,**c**) Scale bar, 1 mm. (**b**,**d**) Lesion size and coverage in the aortic arch (left) and number of lesions in the thoracic aorta (right) of these mice (*n*=3–6). Data are means±s.e.m.; *n* and *P* values are indicated; *P<0.05, unpaired two-tailed *t* test.

Consistent with less substantial changes in the number of circulating inflammatory cells in mice with Mcp1 deletion in endothelial cells, atherosclerosis progressed similarly in these mice and their control littermates: 2 months after feeding the mice with HFD, the number and size of atherosclerotic lesions in the aorta of mice with Mcp1 deletion in endothelial cells remained unchanged (Fig. 2a,b; Supplementary Fig. 4a–c). In contrast—and unexpectedly—, the number and size of aortic atherosclerotic lesions was 2–4-fold decreased in mice lacking Mcp1 in nestin^+^ cells (Fig. 2c,d; Supplementary Fig. 4d–f), despite the increased number of circulating inflammatory cells in these mice. Together, these results indicate that Mcp1 deletion in nestin^+^ cells—but not in endothelial cells only—delays atherogenesis.

### Mcp1 from nestin^+^ cells directs aortic inflammatory infiltration

Consistently with unchanged atheroma progression in mice with Mcp1 deletion in endothelial cells (Fig. 2a,b), the frequency of CD45^+^, CD11b^+^ Ly6G^high^ and CD11b^+^ Ly6C^high^ unchanged, despite a specific 50% reduction in Mcp1 mRNA expression in the aorta of experimental mice (Supplementary Fig. 5).

Previous studies have correlated directly circulating inflammatory cells with atherogenesis^35^. On the contrary, a transient increase in circulating inflammatory following Mcp1 deletion in nestin^+^ cells correlated with delayed atheroma plaque formation in these mice. Therefore, we hypothesized that inflammatory infiltration in the aorta might also depend on Mcp1 produced locally by nestin^+^ cells.

**Figure 3:**
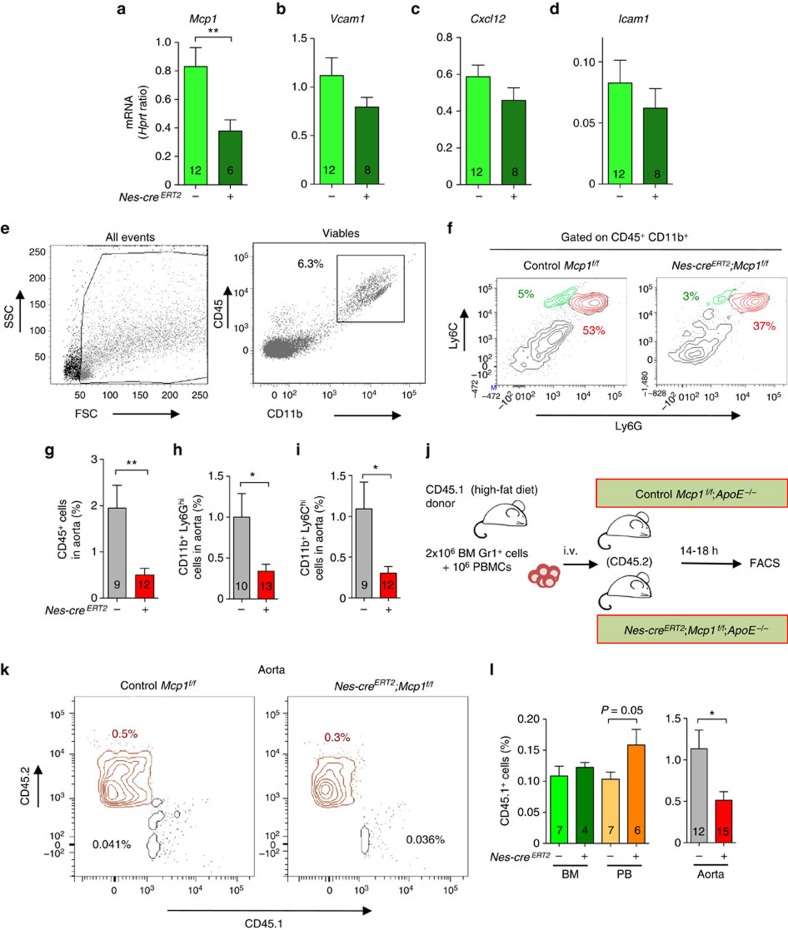
Mcp1 deletion in nestin^+^ cells decreases the inflammatory infiltration in the aortic wall. (**a**–**d**) QPCR analysis of *Mcp1, Cxcl12* and *Icam1* mRNA in aorta from mice fed with chow after the tamoxifen injection (*n*=6–12). (**e**) Flow cytometry plots of aortic cells (left) and myeloid cells (right). (**f**) Representative flow cytometry diagrams of the aortas depicting inflammatory neutrophils (CD11b^+^ Ly6G^high^, red) and inflammatory monocytes (CD11b^+^ Ly6C^high^, green) in both groups of mice. Frequency of (**g**) hematopoietic cells (*n*=9–12), (**h**) inflammatory neutrophils (*n*=10–13) and (**i**) inflammatory monocytes in the aortas of *Nes-cre*^*ERT2*^*;Mcp1*^*f/f*^;*ApoE*^−/−^ mice and control littermates fed with HFD for 2 months (*n*=9–12). (**j**) Design of adoptive transfer experiments. BM Gr1^+^ leukocytes and peripheral blood mononuclear cells (PBMCs) from CD45.1 mice fed with HFD were intravenous transplanted into *Nes-cre*^*ERT2*^*;Mcp1*^*f/f*^;*ApoE*^−/−^ mice and control littermates previously treated with tamoxifen and fed with HFD for 2 months. (**k**) Representative flow cytometry diagrams of CD45.1^+^ and CD45.2^+^ cells in the aortas of *Nes-cre*^*ERT2*^*;Mcp1*^*f/f*^;*ApoE*^−/−^ mice and control littermates. (**l**) Frequency of CD45.1^+^ cells in the BM, the peripheral blood (PB) and the aorta of both groups of mice (*n*=6–15). (**e**,**f**,**k**) The frequencies of the gated populations are indicated. Data are means±s.e.m.; *n* and *P* values are indicated; *P<0.05, unpaired two-tailed *t* test.

Inflammatory infiltration starts with the adhesion and recruitment of circulating inflammatory neutrophils and monocytes through adhesion molecules^1^,^2^. Soon after tamoxifen administration, *Mcp1* mRNA expression—but not *Cxcl12* or *Icam1* expression— was 2-fold reduced in the aorta of mice with *Mcp1* deletion in nestin^+^ cells (Fig. 3a–d). Consistent with unchanged expression of adhesion molecules, the number of rolling leukocytes or their velocity, measured by intravital microscopy of the carotid artery, remained unaffected 3 or 5 weeks after HFD (Supplementary Fig. 6; Supplementary Movies 1 and 2). However, the frequency of CD45^+^ hematopoietic cells, CD11b^+^ Ly6G^high^ inflammatory neutrophils and CD11b^+^ Ly6C^high^ inflammatory monocytes was 3–4-fold reduced in the aortas of *Nes-cre*^*ERT2*^*;Mcp1*^*f/f*^*;ApoE*^*−/−*^ mice, compared with control *Mcp1*^*f/f*^*;ApoE*^*−/−*^ mice that were also treated with tamoxifen and fed with HFD for 2 months (Fig. 3e–i). Therefore, we performed adoptive transfer experiments to determine whether leukocyte migration into the aorta might be compromised in mice lacking Mcp1 in nestin^+^ cells. We transplanted Gr1^+^ BM leukocytes and peripheral blood mononuclear cells, obtained from CD45.1 mice fed with HFD for 2 months, into (CD45.2) *Nes-cre*^*ERT2*^*;Mcp1*^*f/f*^*;ApoE*^*−/−*^ mice and control *Mcp1*^*f/f*^*;ApoE*^*−/−*^ littermates (Fig. 3j). Flow cytometry analyses performed 14–18 h later showed accumulation of CD45.1^+^ cells in the circulation of mice lacking Mcp1 in nestin^+^ cells. This was associated with 2-fold-reduced frequency of CD45.1^+^ cells in the aorta of these mice (Fig. 3k,l). These results suggest that nestin^+^ cells regulate the inflammatory response in atherosclerosis through Mcp1 production in two different compartments. Whereas nestin^+^ BMSCs regulate the egress of inflammatory cells to circulation, nestin^+^ residing in the aortic wall might regulate inflammatory infiltration. Therefore we studied the possible contribution of nestin^+^ cells residing in the artery wall to atheroma plaque formation.

### Nestin^+^ stromal and endothelial cells in the atheroma plaque

**Figure 4:**
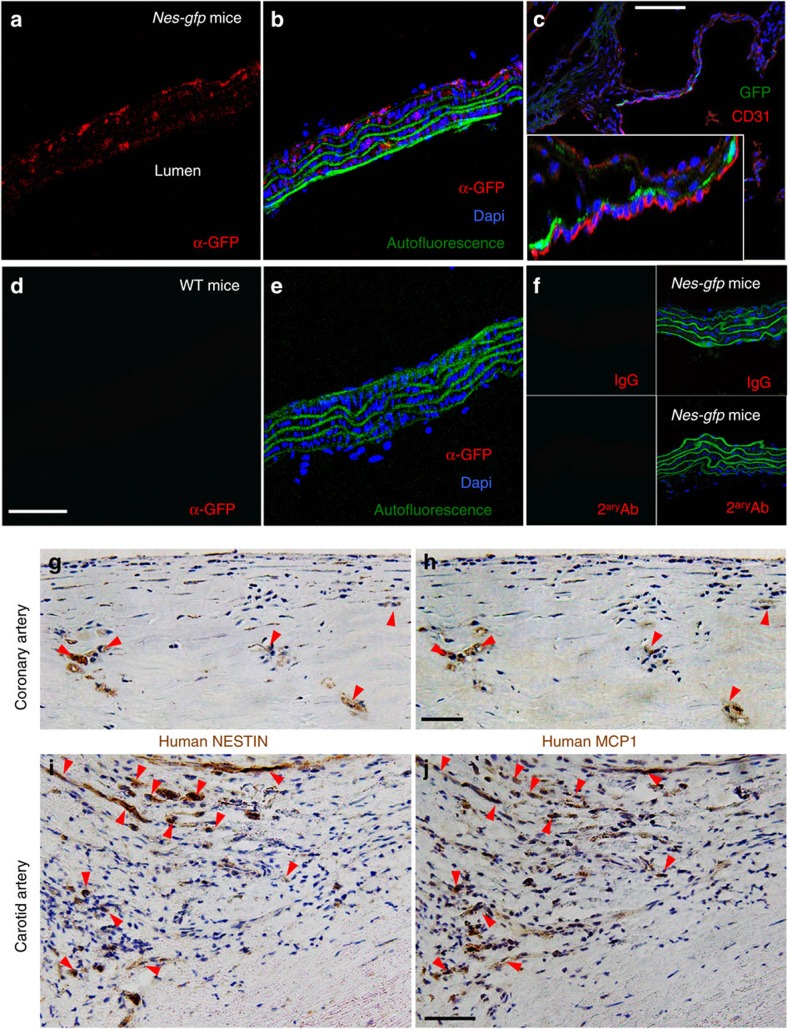
Nestin^+^ cells in the aortic adventitia and media express Mcp1 in mice and humans. Immunofluorescence of sections from (**a**,**b**,**f**) thoracic aorta and (**c**) aortic valves from *Nes-Gfp* mice using antibodies against (**a**,**b**) GFP and (**c**) CD31 (red). Note that nestin^+^ cells are mainly distributed in the adventitia (**b**) or in close proximity of valve endothelial cells (**c**). Inset, high magnification of the aortic leaflets. (**d**,**e**) Immunofluorescence of representative control section from WT thoracic aorta using antibodies against GFP (red). The absence of red signal demonstrates the specificity of the staining. (**b**,**e**) The autofluorescence of the smooth muscle layer is shown in green. (**f**) Negative control immunofluorescence on *Nes-gfp* aorta using control IgG (top panels) and/or secondary antibodies (bottom panels). (**b**,**c**,**e**,**f**) Nuclei were counterstained with DAPI (blue). (**g**–**j**) Immunofluorescence of (**g**–**i**) NESTIN and (**h**–**j**) MCP1 in consecutive cross sections from human (**g**,**h**) coronary and (**i**,**j**) carotid artery samples. Red arrowheads depict cells that express both proteins in consecutive sections. (**a**,**b**,**d**–**f,g**–**j**) Scale bars, 50 μm, (**c**) 100 μm.

Consistent with a previous study^36^, immunofluorescence of aortic sections of *Nes-gfp* mice showed the presence of GFP^+^ cells that were distinct from vascular smooth muscle cells in the media and the adventitia, where they were more abundant (adventitia, 23±6%; media, 8±3%) (Fig. 4a,b). In the aortic valves, Nes-GFP^+^ cells were closely associated with CD31^+^ endothelial cells of the aortic leaflets (Fig. 4c). Control isotype antibodies demonstrated the specificity of anti-GFP antibodies used to circumvent the reportedly high autofluorescence of aortic smooth muscle (Fig. 4d–f). Aortic Nes-GFP^+^ cells were enriched in *Mcp1* and endogenous *Nestin* mRNA expression (Supplementary Fig. 7a). Also, *Mcp1* mRNA levels were much higher in the aorta than in the BM, both in steady state and 5 weeks after HFD (Supplementary Fig. 7b). Recent studies have reported the expression of NESTIN in human blood vessels^37^ and in atheroma plaque neovessels^38^. To further investigate the relationship of MCP1-producing cells with nestin^+^ cells residing in the wall of large human arteries, we performed NESTIN and MCP1 immunohistochemistry in consecutive cross sections from human coronary and carotid arteries. MCP1 and NESTIN expression showed a high degree of overlap in human atherosclerotic arteries (Fig. 4g–j; Supplementary Fig. 7c–f).

**Figure 5:**
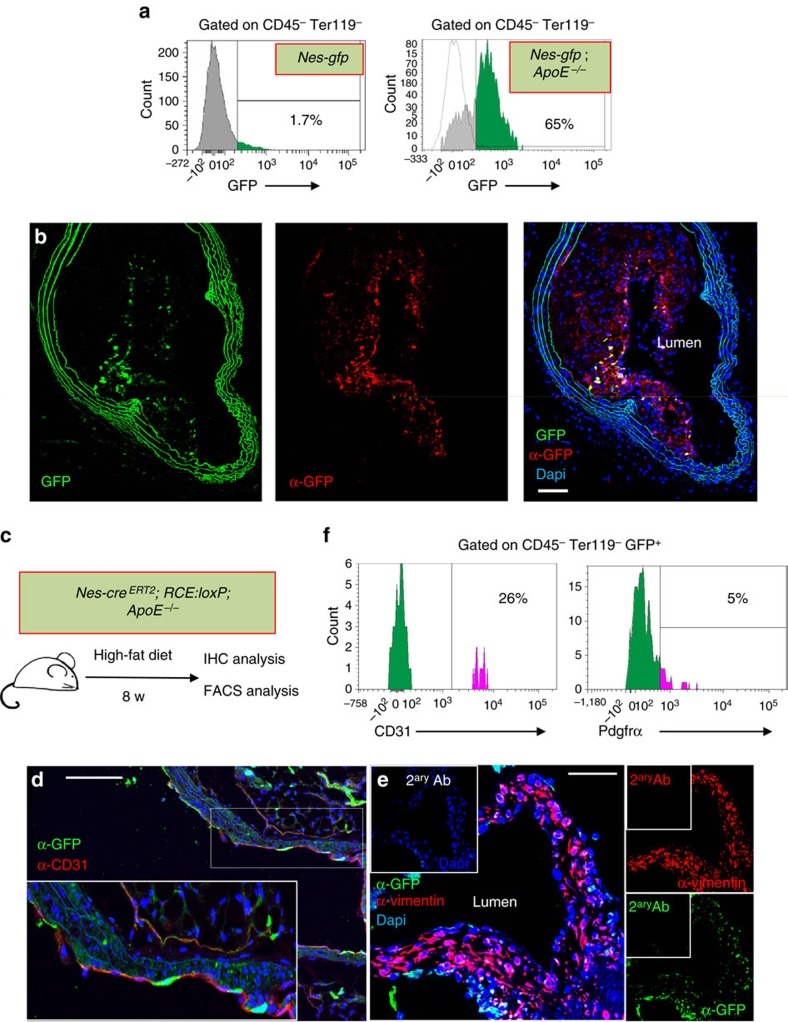
Nestin^+^ cells participate in the formation of the atheroma plaque. (**a**) Flow cytometry of aortic stromal cells showing GFP expression in *Nes-gfp* mice fed with chow diet (left panel) and in *Nes-gfp*;*ApoE*^*−/−*^ mice fed with HFD for 2 months (right panel). (**b**) Representative GFP immunofluorescence of a section of the brachiocephalic artery branching out of the aortic arch of *Nes-Gfp;ApoE*^*−/−*^ mice fed with HFD for 2 months. Abundant GFP^+^ cells were found in the atheroma plaque and the adventitial layer. GFP was detected with an anti-GFP antibody (red). Nuclei were counterstained with DAPI (blue). (**c**) Experimental design of lineage-tracing studies. *Nes-cre*^*ERT2*^*;Rosa26-Gfp;ApoE*^*−/−*^ mice were injected with tamoxifen to trace the progeny of nestin^+^ cells and were fed with HFD for 2 months. (**d**) Immunofluorescence of the aortic valves using anti-GFP and anti-CD31 antibodies. Nuclei were counterstained with DAPI (blue). Inset, higher magnification of aortic leaflet. (**e**) Immunofluorescence of the aortic valves with anti-vimentin antibody. Nuclei were counterstained with DAPI (blue). (**f**) Flow cytometry histogram showing Pdgfrα and CD31 expression in aortic CD45^−^ Ter119^−^ GFP^+^ cells. The frequencies of depicted populations are indicated. (**b**–**e**) Scale bar, 100 μm.

Approximately 20% aortic Nes-GFP^+^ cells were CD45- Sca1^+^ stromal cells (Supplementary Fig. 8)—a cell population with progenitor features previously shown to contribute to atheroma plaque formation^23^. To study the contribution of nestin^+^ cells residing in the artery wall to atherogenesis we intercrossed *Nes-gfp* mice with *ApoE*^−/−^ mice. In adult *Nes-gfp* mice fed with chow diet, only ∼2% of the CD45^−^ Ter119^−^ aortic stromal cells expressed GFP; however, this frequency increased >30-fold in *Nes-gfp;ApoE*^−/−^ mice fed with HFD for two months (Fig. 5a). Immunofluorescence of GFP in brachiocephalic artery sections showed nestin^+^ cells mainly located inside the atheroma plaque and in the fibrous cap (Fig. 5b). We further studied the contribution of nestin^+^ cells to atheroma plaque formation through lineage-tracing experiments using *Nes-Cre*^*ERT2*^;*ApoE*^−/−^ mice intercrossed with *RCE:loxP* mice, which express the R26R CAG-boosted EGFP (RCE) reporter allele harbouring a *loxP*-flanked STOP cassette^39^. We injected *Nes-Cre*^*ERT2*^*;ApoE*^−/−^*;RCE:loxP* mice with tamoxifen and fed them with HFD for 8 weeks (Fig. 5c). Whole-mount immunofluorescence of the aortas showed GFP^+^ cells, different from smooth muscle cells, invading the atheroma plaque from the outer layers of the artery wall (Supplementary Fig. 9a–c; Supplementary Movie 3). Some Nes-Cre^ERT2^-traced cells co-localized with CD31^+^ endothelial cells (Fig. 5d; Supplementary Fig. 9d–I; Supplementary Movie 4), whereas other Nes-Cre^ERT2^-traced cells expressed the mesenchymal marker vimentin (Fig. 5e). Flow cytometry analysis confirmed the contribution of nestin^+^ cells to CD45^−^ Ter119^−^ Pdgfrα^+^ mesenchymal cells and CD45^−^ Ter119^−^ CD31^+^ endothelial cells in the atherosclerotic aorta (Fig. 5f).

To further verify that both nestin^+^ cell derivatives contribute to Mcp1 production in the artery wall, we used a transgenic line expressing GFP under the regulatory elements of the *Mcp1* gene^27^. Endogenous Mcp1 mRNA expression was 15-fold higher in GFP^+^ cells than in GFP^−^ cells isolated from the aorta of these mice (Supplementary Fig. 10a). We intercrossed *Mcp1-gfp* mice with *ApoE*^*−/−*^ mice. Consistent with our previous findings, GFP^+^ cells were detected in the atheroma plaque of *Mcp1-gfp;ApoE*^*−/−*^ mice fed with HFD for 8 weeks (Supplementary Fig. 10b). To further define this stromal contribution, *Mcp1-gfp;ApoE*^*−/−*^ mice were lethally irradiated, transplanted with *ApoE*^−/−^ BM cells and fed with HFD for 8 weeks once recovered from transplantation (Supplementary Fig. 10c). Flow cytometry analysis confirmed the absence of residual monocytes expressing Mcp1-GFP and the presence of GFP^+^ non-hematopoietic stromal cells in the atherosclerotic aorta. Consistent with our lineage-tracing studies using *Nes-Cre*^*ERT2*^ mice, ∼20% Mcp1-GFP^+^ cells expressed CD31 and ∼30% were Vcam1^+^ (Supplementary Fig. 10d–g). Together, these results indicate that, during atherogenesis, nestin^+^ cells increase in the atheroma plaque, where they locally guide inflammatory infiltration through Mcp1 production.

### Delayed calcification in animals lacking Mcp1 in nestin^+^ cells

**Figure 6:**
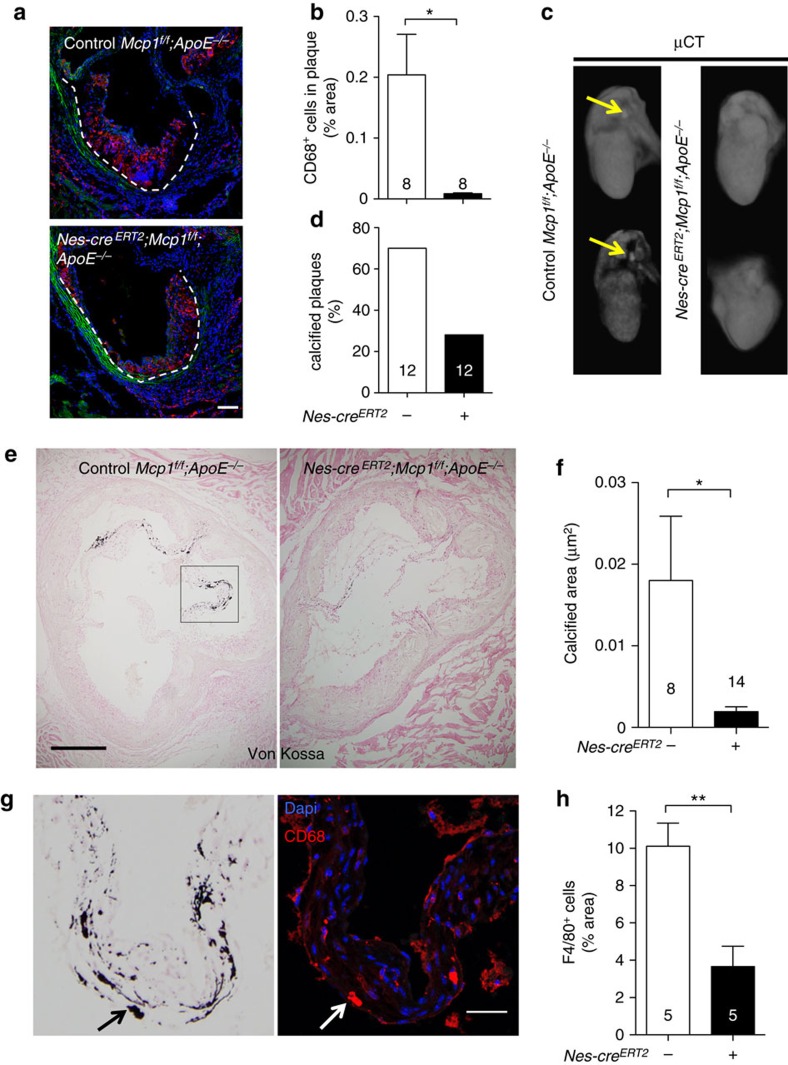
Mcp1 deletion in nestin^+^ cells reduces vascular calcification. (**a**) Immunofluorescence of CD68 (red) to label macrophages infiltrated in the valves of *Nes-cre*^*ERT2*^*;Mcp1*^*f/f*^;*ApoE*^−/−^ mice and *Mcp1*^*f/f*^;*ApoE*^−/−^ controls fed with HFD for 6 weeks. Nuclei were counterstained with DAPI (blue). (**b**) Quantification of CD68^+^ cells in the atheroma plaque of these mice (*n*=8). (**c**) Representative *ex-vivo* microtomography (μCT) photographs of the hearts of *Nes-cre*^*ERT2*^*;Mcp1*^*f/f*^;*ApoE*^−/−^ mice and *Mcp1*^*f/f*^;*ApoE*^−/−^ controls. Arrows indicate calcified plaques. (**d**) Frequency of hearts that showed calcified plaques (*n*=12). (**e**) Representative sections of Von Kossa staining of calcium deposits (black) in the aortic valves of these mice. (**f**) Calcified area in three sections from the aortic valves of these mice (*n*=8–14). (**g**) Consecutive aortic valves sections stained with Von Kossa (black) or DAPI (blue) and anti-CD68 antibodies (red). Note the proximity of CD68^+^ macrophages to the calcified areas (arrow, example). (**h**) Number of F4/80^+^ macrophages (not shown) infiltrated in the aortic leaflets (*n*=5). (**b**,**d**,**f**,**h**) Data are means±s.e.m.; *n* and *P* values are indicated; *P<0.05, **P<0.01, unpaired two-tailed *t* test. (**a**, **g**) Scale bars, 100 μm, (**e**) 200 μm.

At more advanced disease stage, the atheroma plaque frequently undergoes calcification^9^—a process largely influenced by the inflammatory infiltration, both in atherosclerosis and in aortic valve disease^40^,^41^. Consistent with unchanged atheroma progression in mice lacking Mcp1 in endothelial cells (Fig. 2a,b), these mice exhibited unaltered aortic CD68^+^ macrophage infiltration, plaque formation and calcification in the aortic valves (Supplementary Fig. 11). In contrast—consistently with the flow cytometry analysis (Fig. 3g–i)—mice lacking Mcp1 in nestin^+^ cells exhibited delayed aortic infiltration by CD68^+^ macrophages, correlated with retarded collagen deposition in the aortic valves of mice lacking Mcp1 in nestin^+^ cells (Fig. 6a,b; Supplementary Fig. 12a,b). We measured plaque calcification by *ex-vivo* microtomography (μCT) of the hearts and aortas of *Nes-cre*^*ERT2*^*;Mcp1*^*f/f*^;*ApoE*^−/−^ mice and *Mcp1*^*f/f*^;*ApoE*^−/−^ controls fed with HFD for 8–12 weeks. Whereas 70% of control mice showed macroscopic calcification, this was detectable only in 30% of the mice lacking Mcp1 in nestin^+^ cells (Fig. 6c,d). Calcium deposits stained with Von Kossa were more abundant in the aortic leaflets. Notably, the calcified area of the aortic valves was 20-fold reduced in mice lacking Mcp1 in nestin^+^ cells (Fig. 6e,f). This was associated with ∼3-fold reduced CD68^+^ macrophages, frequently localized near calcified areas of the aortic leaflets (Fig. 6g,h), where Mcp1-expressing stromal cells were also found (Supplementary Fig. 12c–f).

Altogether, these results suggest that nestin^+^ stromal cells regulate inflammatory infiltration in the atherosclerotic plaque through Mcp1 production and point towards a crosstalk between inflammatory cells and stromal cells during atheroma plaque formation.

## Discussion

Several studies support the idea that atherosclerosis is a consequence of imbalanced lipid metabolism and a maladaptive immune response. However, the mechanisms that direct inflammatory migration from the lymphoid organs to the aorta—where they contribute critically to the atheroma plaque formation—are not fully understood. Particularly, whether stromal cells regulate inflammatory cell traffic in chronic inflammatory diseases (such as atherosclerosis) and/or participate in atherosclerotic plaque formation has remained unknown. The present study demonstrates that nestin^+^ stromal cells direct inflammatory cell migration in different compartments as a critical initial step in atherosclerosis. It also suggests that Mcp1 expression by stromal cells residing in different organs is a gatekeeper, directing the traffic of inflammatory cells during chronic inflammation.

A recent report has suggested that nestin^+^ cells found in the adventitia layer are multipotent^36^. Our results in *ApoE*^−/−^ mice fed with HFD show that BM nestin^+^ cells regulate the egress of inflammatory monocytes and neutrophils. In the aorta of these mice, nestin^+^ stromal cells, residing mainly in the adventitia, increased ∼30 times during atherogenesis and contributed to endothelial cells in the atherosclerotic plaque. Mcp1 deletion in nestin^+^ cells—but not in endothelial cells only—increased circulating pro-inflammatory monocytes and neutrophils, but decreased their aortic infiltration (without affecting leukocyte adhesion to endothelium), delayed atheroma plaque formation and aortic valve calcification. Therefore, these results suggest that nestin expression marks stromal and endothelial cells that systemically regulate inflammatory cell migration in atherosclerosis.

Interestingly, this side-by-side comparison of the specific contribution of endothelial and stromal cells has uncovered important differences between acute and chronic inflammation. Previous studies showed that, in acute inflammation, Mcp1 produced by both endothelial cells and mesenchymal progenitors is required to redistribute inflammatory monocytes in the BM (during minutes-hours after lipopolysaccharide (LPS) administration/infection), and facilitate their mobilization in response to pathogens^27^. This seems to be different during chronic inflammation, since the present study shows that Mcp1 deletion in endothelial and mesenchymal cells differentially impacts the traffic of inflammatory cells. Mcp1 deletion in mesenchymal progenitors does not prevent this mobilization in atherosclerosis but, instead, it increases circulating inflammatory cells. It is important to note that both studies have markedly distinct kinetics (from minutes-hours to weeks-months) and different inflammatory stimuli (LPS injection versus high level of blood cholesterol content in *ApoE*^*−/−*^ mice fed with HFD). Therefore, the response of endothelial and stromal cells might depend on the nature of the inflammatory stimuli and the role of these cells might also vary during acute and chronic inflammation. Our results in atherosclerosis are consistent with the overall chemotactic role of Mcp1 in various compartments and point towards a gatekeeper function of this chemokine expressed by nestin^+^ cells.

We previously showed that sympathetic efferent activity in the BM regulates the traffic of hematopoietic stem cells by activating β_3_-adrenergic receptors expressed in BMSCs^26^,^42^. Following myocardial infarction, this mechanism has been shown to relocate hematopoietic progenitors from the BM to the spleen as a source of inflammatory cells that aggravate atherosclerosis^43^. In our study, Mcp1 produced by nestin^+^ cells seem to preferentially regulate the migration of inflammatory cells in the BM and the aorta, rather than in the spleen. A very recent study has identified CCR2^+^ hematopoietic progenitor cells as the main primitive population mobilized in this context^44^. It would be interesting to determine in future studies whether nestin^+^ cells regulate the migration of CCR2^+^ HSPCs during chronic inflammation.

We have shown here that nestin^+^ cells in different tissues direct inflammatory cell migration through Mcp1 production: in the BM they regulate the traffic of inflammatory cells towards circulation, whereas in the artery wall they direct inflammatory infiltration. It is also important to note that chemokines typically function in short distance ranges and therefore, not only the amount of Mcp1 produced, but also the source location might importantly determine monocyte attraction and migration. Moreover, the mechanisms underlying Mcp1-regulated traffic of inflammatory cells are not yet fully elucidated, with different possible non-exclusive mechanisms^45^.

To our knowledge, our study also represents the first example of high circulating inflammatory cells inversely (instead of directly) correlating with atherosclerosis burden. In previous studies, increased circulating inflammatory cells (monocytosis) have invariably correlated directly with atherosclerosis progression^34^,^35^,^46^. In contrast, a transient increase in circulating inflammatory cells did not promote atherosclerosis in our study but, on the contrary, it delayed atheroma plaque formation. A likely explanation is that the most relevant source of Mcp1 in atherosclerosis is the nestin^+^ cell population residing in the aorta. This contention is supported by the following data: (a) Mcp1 expression levels were higher in the aorta than in the BM, both in steady state and after 2 months in HFD; (b) nestin^+^ cells are the main source of Mcp1 in the aorta and this aortic cell population expands by 30-fold during atherogenesis; (c) Mcp1 deletion in nestin^+^ cells decreased inflammatory infiltration in the aorta; (d) Mcp1 deletion in nestin^+^ cells quickly reduces the levels of the chemokine in the aorta. Although these differences disappear after 2 months in HFD (probably because other Mcp1-producing cells, like monocytes, accumulate in the plaque), they are sufficient to cause a marked delay in atherosclerosis progression.

Inflammation can compromise the integrity of endothelial cells in vasa vasorum^14^, contributing to plaque haemorrhage^18^,^47^,^48^. One study has shown that vasa vasorum expansion precedes vessel wall thickening and plaque development^49^, whereas others have suggested that inflammatory infiltration starts in the adventitia layer, and later extends to the intima^11^,^12^. Several studies have found vascular progenitor cells residing in the adventitia. Among them, multipotent pericytes associated with microvessels can differentiate into mesodermal and endodermal cell lineages, including smooth muscle cells and fibroblasts^23^,^50^. Tissue resident endothelial progenitors have also been detected in different blood vessels^51^,^52^. Our findings are consistent with these studies, since nestin^+^ cells in the artery wall expanded dramatically in *ApoE*^*−/−*^ mice fed with HFD and included both mesenchymal stromal cells and endothelial cells integrated in these neovessels. Whether both lineages are derived from common or different progenitors remains to be determined. Overall, our side-by-side comparison suggests that Mcp1 production by the stromal cells (rather than endothelial cells) is key in directing inflammatory cell traffic in atherosclerosis.

Monocyte infiltration can trigger plaque calcification^53^,^54^ and disruption of the Mcp1–Ccr2 axis reduces calcification in human carotid arteries^55^. Therefore, the reduced aortic inflammatory infiltration in mice lacking Mcp1 in nestin^+^ cells might explain also the decreased vascular calcification in these mice as a result of the overall delay in the atherosclerotic process in these mice. In support of this possibility, we found calcium deposits in close proximity of inflammatory cells in the aortic leaflets. Of note, MCP1 and NESTIN expression largely overlapped in human atherosclerotic arteries, suggesting that similar mechanisms might take place in humans.

Altogether, this study highlights the relevance of nestin^+^ cells in directing inflammatory cell migration during the early stages of atherosclerosis, possibly applicable to other chronic inflammatory diseases.

## Methods

### Mice

Animals used in this study included *Mcp1*^*f/f*^ (ref. 27) *Mcp1-gfp*^27^, *Nes-gfp*^31^, *Nes-cre*^*ERT2*^ (ref. 32), *RCE:loxP* (ref. 39), *Cdh5-cre*^*ERT2*^ (ref. 33), *ApoE*^*−/−*^(ref. 4) and CD45.1 and CD45.2 C57BL/6 mice (Jackson Laboratories). All the mice were bred in specific pathogen-free facilities. Mice of both genders (sex-matched) from 6 to 8 weeks of age were fed with atherogenic high-fat diet (HFD: 10, 8% total fat, 0.75% cholesterol; S8492, Ssniff, Germany) for the indicated time periods. Compound *NesCre*^*ERT2*^ mice, *Cdh5-cre*^*ERT2*^ mice and control littermate mice were injected with tamoxifen (140 mg kg^−1^ in corn oil, intraperitoneal) three times on alternate days. The treatment was repeated monthly. Experimental procedures were approved by the Animal Care and Use Committees of the Spanish National Cardiovascular Research Center and Comunidad Autónoma de Madrid (PA-47/11 and ES280790000176; Real Decreto RD53/2013). Mice were genotyped using proteinase K at 10 mg ml^−1^ in proteinase K buffer (100 mM Tris–HCl, pH 8, 100 mM EDTA, 250 mM NaCl) 3 h at 55 °C. Genomic DNA were extracted precipitating with isopropanol. DNA was washed with ethanol 70% and resuspended in TE buffer. 1 μl was used to the PCR. The sequences of the primer pairs used are listed in the Supplementary Table 1.

### Irradiation and transplantation

Lethally irradiated (137Cs source, 12 Gy whole body irradiation, split dose 6+6 Gy, 3 h apart) mice were transplanted the same day with 2 × 10^6^ BM nucleated cells from adult donor mice. Animals were allowed to recover from transplantation for one week, after which they were fed with HFD for 8 weeks.

### Imaging

Confocal images of fluorescent signals were acquired with a laser scanning confocal microscope (Zeiss LSM 700, 25 × /0.85 or Leica SP5 20 × /0.7, 40 × /1.25, × 63/1.4). Optical *z*-stack projections were generated with the Zen2011 software package (Zeiss, Germany) using a maximal intensity algorithm or with LAS software for Leica images. For quantification, images were post- processed and analyzed using ImageJ^56^.

### μCT analyses

*Ex-vivo* CT imaging of whole hearts connected to the thoracic aorta was performed using a nanoPET/CT small-animal imaging system (Bioscan, Washington DC, USA) equipped with an micro-focus X-ray source and a high-resolution radiation-imaging device featuring a 1,024 × 3,596 pixel photodiode array with 48 μm pixel pitch. For CT measurements, scan parameters were 360 projections per rotations, 45 kV (peak) 145 μA current, and a detector pixel size of 71 μm. Acquisition and reconstruction were performed with proprietary Nucline software (Mediso, Budapest, Hungary).

### Real-time RT-PCR

The mRNA was extracted from cells isolated by FACS using the Dynabead mRNA Direct kit (Invitrogen). Whole aorta tissue was homogenized using a tissue-lyser machine (Qiagen). Briefly, samples were frozen with liquid nitrogen and were homogenized with the tissue-lysser for 2 min and frozen again in liquid nitrogen. This procedure was repeated four times. Whole aorta RNA was extracted using Tri Reagent (Sigma-Aldrich), followed by RNA purification with the RNeasy mini kit (Qiagen). Reverse transcription of 500 ng RNA was performed using the High-Capacity cDNA Reverse Transcription kit (Applied Biosystems). QPCR experiments were conducted using SYBR Green (Applied Biosystems). Samples were loaded on a 384-well plate and analyzed on a 7900HT machine (Applied Biosystems). The expression level of each gene was determined using the relative standard curve method. The standard curve was performed using serial dilutions of mouse reference total RNA (Clontech). The expression level of each gene was calculated by interpolation from the standard curve. All values were normalized with *Gapdh* and *Hprt1* as endogenous housekeeping genes. The sequences of the primer pairs used are listed in the Supplementary Table 1.

### Histology

Maximov's hematoxylin staining was performed in deparaffinized sections after re-hydration. Nuclei were stained with the Mayer's hematoxylin solution for 15 min, followed by overnight incubation in eosin Y/Azur II solution for cytoplasm staining. After rinsing briefly in distilled water, sections were quickly dehydrated in ethanol (70, 95 and 100%) and xylene. Slides were mounted in DPX.

Thricromic staining of the Masson protocol was used for atherosclerotic plaque visualization. Cryopreserved sections were washed with PBS and fixed in Bouin solution for 1 h, washed with tap water (5 min, twice) and stained with weigert's hematoxylin for 8 min, tap water washed and a dipped in acidified water, followed by staining with Biebrich–Scarlet solution for 7 min. After washing with distilled water, we incubated the sections for 5–10 min in phosphomolybdic acid 5% and washed them with distilled water. The sections were impregnated in fast green or aniline blue for 2 min, dehydrated and mounted in DPX.

For Oil Red O staining, whole-mount aortas were fixed with 2% paraformaldehyde o/n at 4 °C and post-fixed twice with 78% methanol for 5 min. Next, the aortas were incubated in 0.2% Oil Red O for 1 h at room temperature (RT) in a shaker. After washing twice with 78% methanol for 5 min, the aortas were mounted on agar plates using mini-pins. Images were acquired using a stereomicroscope (Olympus). The extent of atherosclerosis was assessed in the aortic roots and the thoracic aorta by quantifying the number and the size of lipid Oil Red O^+^ lipids deposits.

For Von Kossa staining, cryopreserved sections were washed with PBS for 5 min, oxidized with 0.2% aqueous potassium permanganate for 30 min, washed with water and bleached in 5% aqueous oxalic acid until they became white. The sections were then incubated in 1% silver nitrate and exposed to a strong light source for ∼1 h, until calcium deposits became dark brown or black. We washed the sections 3 times with distilled water and incubated them with 2.5% sodium thiosulfate for 5 min. After several washes with distilled water, sections were counterstained with 1% safranin O or 0.1% nuclear fast red for 1 min, dehydrated and mounted with DPX.

### Immunofluorescence and inmunohistochemistry

For immunofluorescence analyses, aorta sections, aortic valve sections or whole-mount aortas were stained following standard procedures. Briefly, cryopreserved sections were washed with PBS for 5 min. A blocking and permeabilization step was performed with TNB solution (100 mM Tris–HCl, 150 mM NaCl, 0.5% blocking reagent (Perkin Elmer)) containing 0.05% Tween for 1 h. The sections were then incubated with different primary antibodies: live-colors rabbit anti-GFP antibody (Biolegend, San Diego, CA, USA), goat anti-GFP antibody (Abcam), antibodies against CD31 (BD Biosciences), CD68 (AbD Serotec, bionova), F4/80 (AbD Serotec, bionova), Ki67 (Abcam) all of them used at 1:100 dilution in TNB buffer o/n at 4 °C. For the anti-vimentin antibody (Cell signaling) the dilution used was 1:200. Following washes with 0.05% Tween in PBS, we incubated the sections with secondary antibodies conjugated to Alexa555, Alexa488 (Molecular Probes, Eugene, OR, USA), or with Cy3-conjugated anti-smooth muscle actin antibody (Sigma) at 1:200 dilution in TNB for 2 h at RT. Following washes with 0.05% Tween in PBS, the sections were incubated with 4′,6-diamidino-2-phenylindole (DAPI) (1:1,000 dilution of 5 mg ml^−1^ stock) for 10 min at RT and covered with mounting medium (Dako). Whole-mount aortas were blocked and permeabilized with 0.1% triton in TNB buffer o/n at 4 °C. After several washes with 0.1% triton in PBS, the aortas were incubated with different primary antibodies at least for 24 h at 4 °C in TNB buffer. Following washes, whole-mount aortas were incubated with secondary antibodies for 3 h at RT and covered with Mounting Medium (Dako).

For inmunohistochemistry, coronary and carotid arteries were collected from freshly excised hearts during transplant operations and by endarterectomy respectively at the Hospital de la Santa Creu i Sant Pau (HSCSP, Barcelona, Spain). The study was approved by HSCSP Ethics Committee (12/007/1292) and was conducted according to the Declaration of Helsinki. Written informed consent was obtained from each patient. Specimens for immunohistochemical studies were fixed overnight in 4% paraformaldehyde/0.1 M PBS (pH 7.4), embedded in paraffin and cut into 3-μm-thick sections with a microtome (Jung RM2055, Leica). Sections were incubated with antibodies against Nestin (1:100; OBT1610, AbD Serotec) or MCP1 (1:50; ab9669, Abcam) in a Dako Autostainer Link 48 using the Dako EnVision Flex kit and the EnVision FLEX Target Retrieval Solution, Low pH. Colour was developed using 3,3′-diaminobenzidine (DAB) and sections were counterstained with haematoxylin. Negative controls, in which the primary antibody was omitted, were included to test for non-specific binding.

### Flow cytometry and fluorescence-activated cell sorting

Bones (limbs, sternum and spine) were crushed in a mortar, filtered through a 40-μm mesh to obtain single cell suspensions, and depleted of red blood cells by lysis in 0.15 M NH_4_Cl for 10 min at 4 °C. Spleen samples were homogenized and filtered prior to lysis; blood samples were directly lysed. Cells (1–2 × 10^6^ cells per sample) were incubated with the appropriate dilution (2–5 μg ml^−1^) of fluorescent antibody conjugates and DAPI for dead cell exclusion, and analyzed on FACS Canto II flow cytometer (BD Biosciences, Franklin Lakes, NJ, USA). Data were analyzed with DIVA software (BD Biosciences, Franklin Lakes, NJ, USA). The following antibody conjugates were used: CD45-PE (30-F11) (1:200), CD45.1-PE-Cy7 (A20) (1:200), CD45.2-APC-Cy7 (104) (1:100), CD11b-Alexa647 (M1/70) (1:200), Ly6C-FITC (AL-21) (1:200), Ly6G-PE (1A8) (1:200), Sca1-PE (E13-161.7) (1:200), CD31-APC (MEC13.3) (1:100), CD140a-APC (APA5) (1:100), Ter119-PE-Cy7 (1:200), Gr1-PE (RB6-85C) (1:200), VCAM1-PE (429) (1:200). When needed, biotinylated antibodies were detected with APC-Cy7-conjugated streptavidin (BD Biosciences) (1:100).

For BM cell sorting, bones (limbs, hips and sternum) were crushed in a mortar and incubated with collagenase I (1 mg ml^−1^) (Sigma) for 1 h at 37 °C with gentle agitation. Collagenase I was inactivated with 2% FBS in PBS and the solution was filtered through a 40-μm mesh to obtain single cell suspensions. Red blood cells were lysed in 0.15 M NH_4_Cl for 10 min at 4 °C. Cells were stained with CD45 (1:200), Ter119 (1:200) and CD31 (1:100) antibodies. Populations of interest were separated in a FACS Aria cell sorter (BD Bioscience). For flow cytometry of mouse aorta, several aortas were pooled, cut in small pieces and digested with 0.25 mg ml^−1^ liberase (Roche) in RPMI1640+10% FBS medium at 37 °C for 1 h with gentle agitation (300 r.p.m.). After this period we inactivated the liberase with 2% FBS in PBS solution. The suspension was filtered through a 40-μm mesh to obtain single cell suspensions that were directly stained for flow cytometry analysis.

### Intravital microscopy

Intravital microscopy was performed in the carotid artery of live animals as previously described^57^. Briefly, mice were anesthetized and were injected with 50 μg of rhodamine 6G (Sigma) that labels mononuclear peripheral blood cells. After, mice were placed in decubitus position, and the carotid artery exposed and carefully dissected. The bifurcation of the common left carotid artery is susceptible to atherosclerosis in humans and mice and is easily accessible with minor surgery. High-resolution *in vivo* images were acquired with this procedure that allows vessel stabilization without significantly compromising blood flow. Images were captured using a 40 × NA 1.0 objective and the CoolSnap camera with 4 × 4 binning in Cy3 channel (1.7 Hz). The number of rolling cells was determined by counting the number of leukocytes crossing an imaginary line perpendicular to the vessel during at least 30 s. Several fields (typically 5) were imaged from the carotid bifurcation, or from the common carotid artery, and each data point represents one field. Although the appearance of plaques in the carotid artery is heterogeneous, for visualization we chose areas prone to plaque development. Data were normalized to the number of rolling cells per minute. Rolling velocity (in μm s^−1^) was determined by measuring time and distances travelled (∼70 μm) by individual leukocytes using a digital caliper with the Slidebook software, (Intelligent Imaging Innovations), and is shown as μm s^−1^. Five to ten leukocytes were analyzed per field and each data point represents one leukocyte.

### Data availability

The authors declare that the data supporting the findings of this study are available within the article and its Supplementary information files.

## Additional information

**How to cite this article:** del Toro, R. *et al*. Nestin^+^ cells direct inflammatory cell migration in atherosclerosis. *Nat. Commun.* 7:12706 doi: 10.1038/ncomms12706 (2016).

## Supplementary Material

Supplementary Information Supplementary Figure 1-12

Supplementary Movie 1 Intravital microscopy showing rhodamine-labelled leukocytes rolling in the common carotid artery of a representative control Mcp1^f/f^;ApoE^-/-^ mouse fed with HFD for 5 weeks.

Supplementary Movie 2 Intravital microscopy showing rhodamine-labelled leukocytes rolling in the common carotid artery of a representative Nes-creERT2;Mcp1^f/f^;ApoE^-/-^ mouse treated with tamoxifen and fed with HFD for 5 weeks.

Supplementary Movie 3 Sequential stack film from a whole mount immunostaining of an atheroma plaque using anti-GFP (far-red channel shown in green pseudocolor) and anti-smooth muscle actin (SMA, red) antibodies. Confocal adquisition was acquire with 20x magnification objective. 14 planes are shown.

Supplementary Movie 4 Sequential stack film from a whole mount immunostaining of an atheroma plaque using anti-GFP (far-red channel shown in green pseudocolor) and CD31 (red) antibodies. Confocal acquisition was with a 20x magnification objective. 10 planes are shown.
