# Supplementary material for: Nestin+ cells direct inflammatory cell migration in atherosclerosis
Source: Nat Commun. 2016 Sep 2;7:12706. doi: 10.1038/ncomms12706 (PMC5025806; doi:10.1038/ncomms12706)
Supplement: Supplementary Information — Supplementary Figure 1-12 [file ncomms12706-s1.pdf]

Supplementary Figure 1

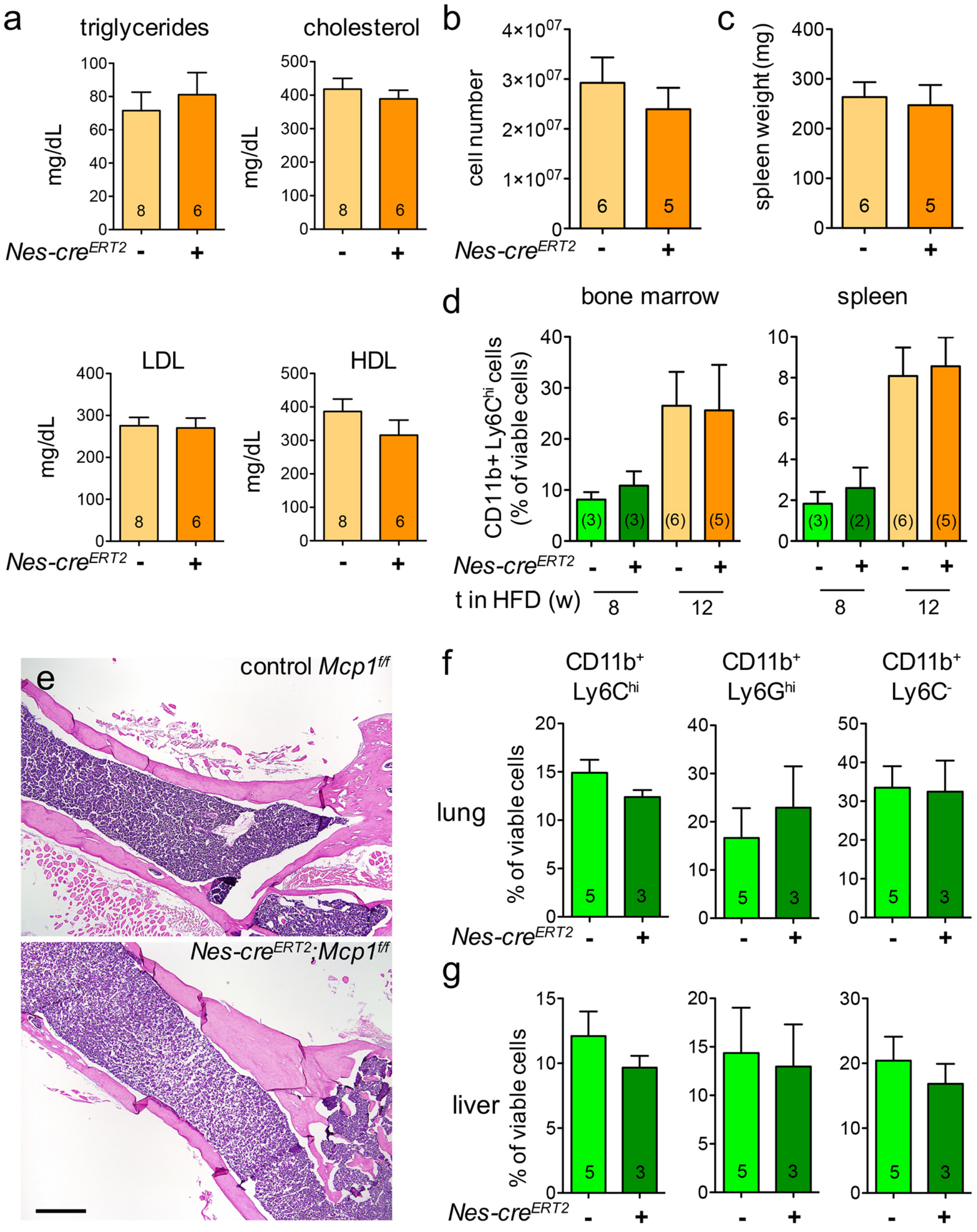

Supplementary Figure 1. Mcp1 deletion in nestin$^+$ cells does not affect BM or spleen inflammatory cell numbers and it does not alter the hyperlipidemia of *ApoE$^{-/-}$* mice. (**a**) Plasma concentration of triglycerides, cholesterol, low- (LDL) and high-density lipoproteins (HDL) in tamoxifen-treated *Nes-cre^{ERT2};Mcp1$^{f/f}$;ApoE$^{-/-}$* mice and *Mcp1$^{f/f}$;ApoE$^{-/-}$* controls mice sacrificed after 8-12 weeks in HFD (*n* = 6-8). (**b**) Nucleated cell number in bone marrow (per four limbs, sternum and spine) (*n* = 5-6). (**c**) Spleen weight (*n* = 5-6). (**d**) Frequencies of bone marrow and spleen inflammatory monocytes and neutrophils in tamoxifen-treated *Nes-Cre^{ERT2};Mcp1$^{f/f}$;ApoE$^{-/-}$* mice and control littermates fed with HFD for 8 and 12 weeks. (**e**) Representative femoral bone marrow sections from these mice (*n* = 2-6). (**f-g**) Frequencies of (**f**) lung and (**g**) liver inflammatory monocytes, inflammatory neutrophils and classical monocytes in tamoxifen-treated *Nes-Cre^{ERT2};Mcp1$^{f/f}$;ApoE$^{-/-}$* mice and control littermates fed with HFD for 8 weeks (*n* = 3-5). *n* indicated in bars. Scale bar, 500 μm. (**e**). (**a-d**, **f-g**) Data are means ± SEM; *n* values are indicated inside bars.

Supplementary Figure 2

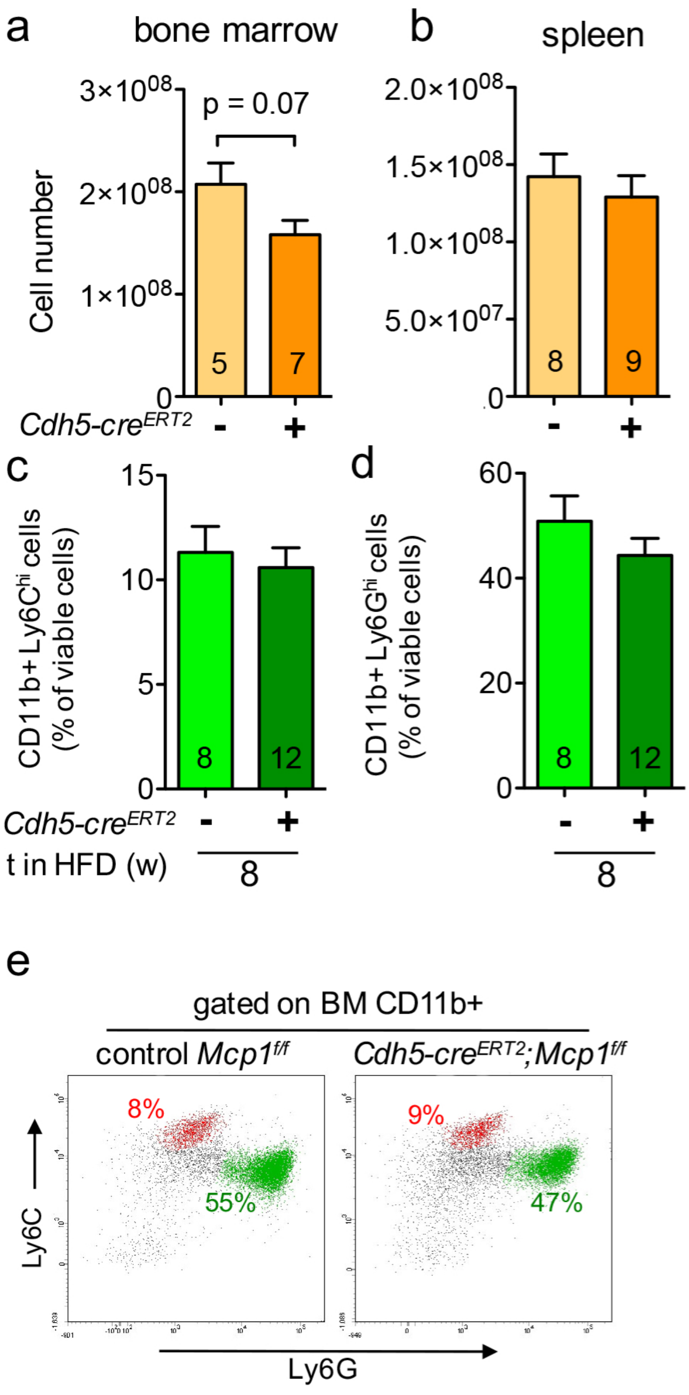

Supplementary Figure 2. Mcp1 deletion in endothelial cells does not alter BM or spleen inflammatory cell numbers. (**a**-**b**) Nucleated cell number in BM (per four limbs, sternum and spine) and spleen (n = 5-9). (**c**-**d**) Frequency of BM (**c**) inflammatory monocytes and (**d**) inflammatory neutrophils in tamoxifen-treated *Cdh5-Cre*^{ERT2};*Mcp1*$^{f/f}$;*ApoE*$^{-/-}$ mice and control littermates fed with HFD for 8 weeks (n = 8-12). (**a**-**d**) Data are means ± SEM; n and p values are indicated; unpaired two-tailed t test. (**e**) Representative flow cytometry diagrams of the bone marrow cells depicting inflammatory neutrophils (CD11b^{+} Ly6G^{high}, green) and inflammatory monocytes (CD11b^{+} Ly6C^{high}, red) in both groups of mice.

Supplementary Figure 3

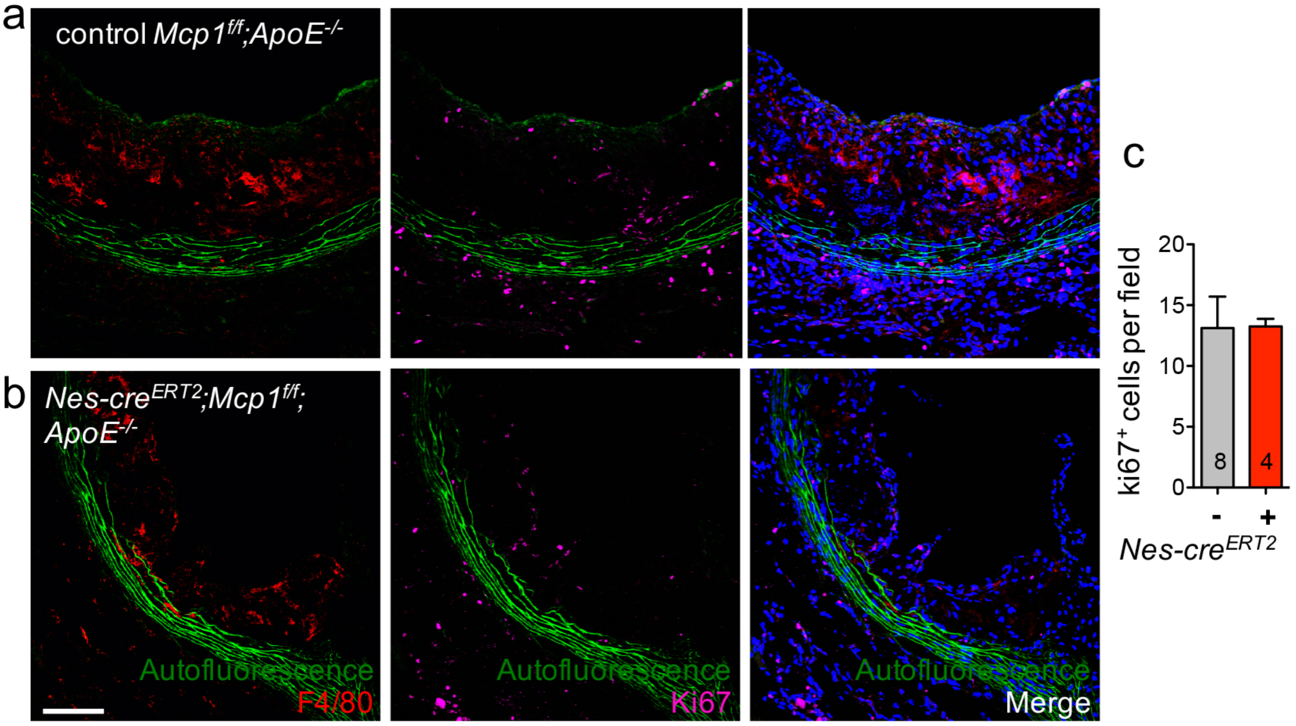

Supplementary Figure 3. Mcp1 deletion in nestin$^+$ cells does not change cell proliferation in the atheroma plaque. (**a**-**b**) Immunofluorescence of sections from aortic valves from (**a**) *Nes-cre^{ERT2};Mcp1$^{f/f}$;ApoE$^{-/-}$* mice and (**b**) *Mcp1$^{f/f}$;ApoE$^{-/-}$* control mice using antibodies to mark macrophages (F4/80, red) and proliferative cells (Ki67, pink). The autofluorescence of the tissue is represented in the green channel. Nuclei were counterstained with Dapi (blue). Scale bar, 100 µm. (**c**) Number of positive Ki67+ in the atheroma plaque was quantified (*n* = 4-8). Data are means ± SEM; *n* values are indicated inside bars.

Supplementary Figure 4

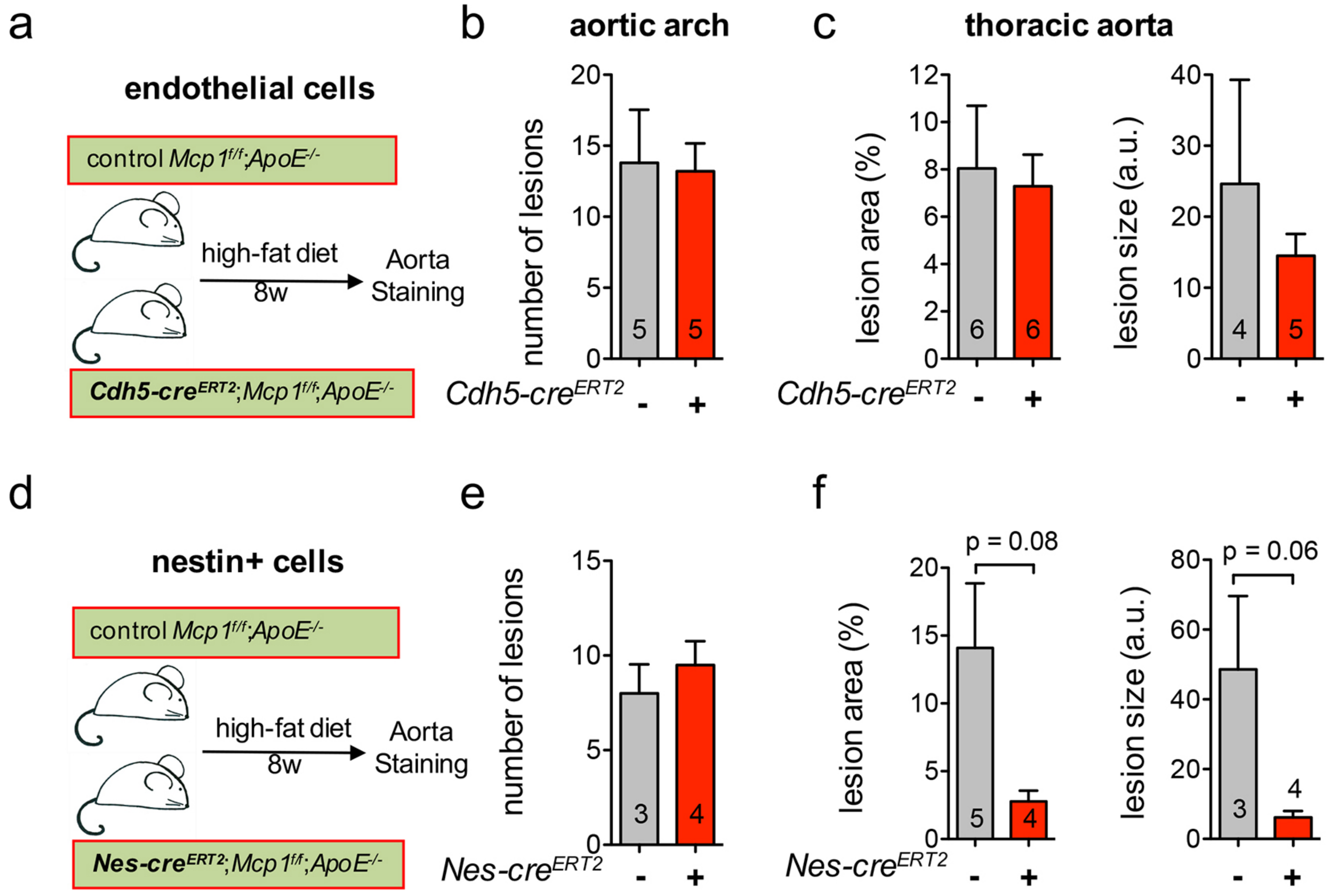

Supplementary Figure 4. Mcp1 deletion in nestin+ cells delays atherosclerosis progression. (**a**, **d**) Experimental design used to study the impact of Mcp1 deletion in (**b**-**c**) endothelial cells and in (**e**-**f**) nestin^{+} cells on atherosclerosis progression. (**b**, **e**) Number of atherosclerotic lesions in the aortic arch, and (**c**, **f**) size and coverage of the lesions in the thoracic aorta of (**b**-**c**) *Cdh5-cre*^{ERT2}*;Mcp1*$^{f/f}$*;ApoE*$^{-/-}$ mice, (**e**-**f**) *Nes-cre*^{ERT2}*;Mcp1*$^{f/f}$*;ApoE*$^{-/-}$ mice and *Mcp1*$^{f/f}$*;ApoE*$^{-/-}$ controls (n = 3-6). Data are means ± SEM; *n* and *p* values are indicated; * $p < 0.05$, unpaired two-tailed *t* test.

Supplementary Figure 5

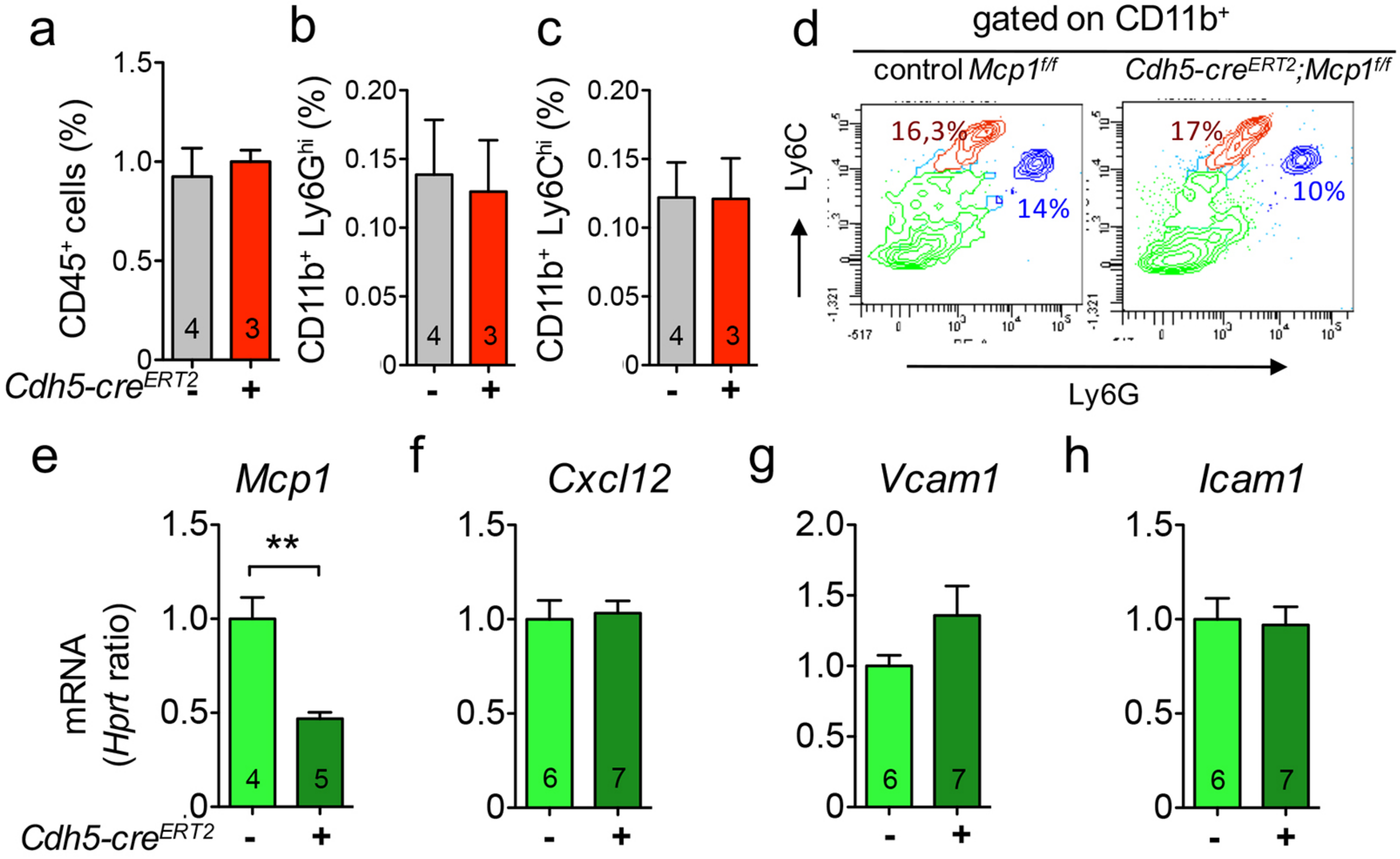

Supplementary Figure 5. Mcp1 deletion in endothelial cells does not change the inflammatory infiltration in the aortic wall. (**a**-**c**) Frequency of (**a**) hematopoietic cells, (**b**) inflammatory neutrophils and (**c**) inflammatory monocytes in the aortas of *Cdh5-cre*^{ERT2};*Mcp1*$^{f/f}$;*ApoE*$^{-/-}$ mice and control littermates fed with HFD for 2 months (n = 3-4). (**d**) Representative flow cytometry diagrams of the aortas depicting inflammatory neutrophils (CD11b^{+} Ly6G^{high}, blue) and monocytes (CD11b^{+} Ly6C^{high}, red) in both groups of mice. (**e**-**h**) QPCR analysis of *Mcp1, Cxcl12*, *Vcam1* and *Icam* mRNA in the aortas of *Cdh5-cre*^{ERT2};*Mcp1*$^{f/f}$;*ApoE*$^{-/-}$ mice and control littermates fed with HFD for 2 months (n = 4-7). Data are means ± SEM; n values are indicated; ** p < 0.01, unpaired two-tailed t test.

Supplementary Figure 6

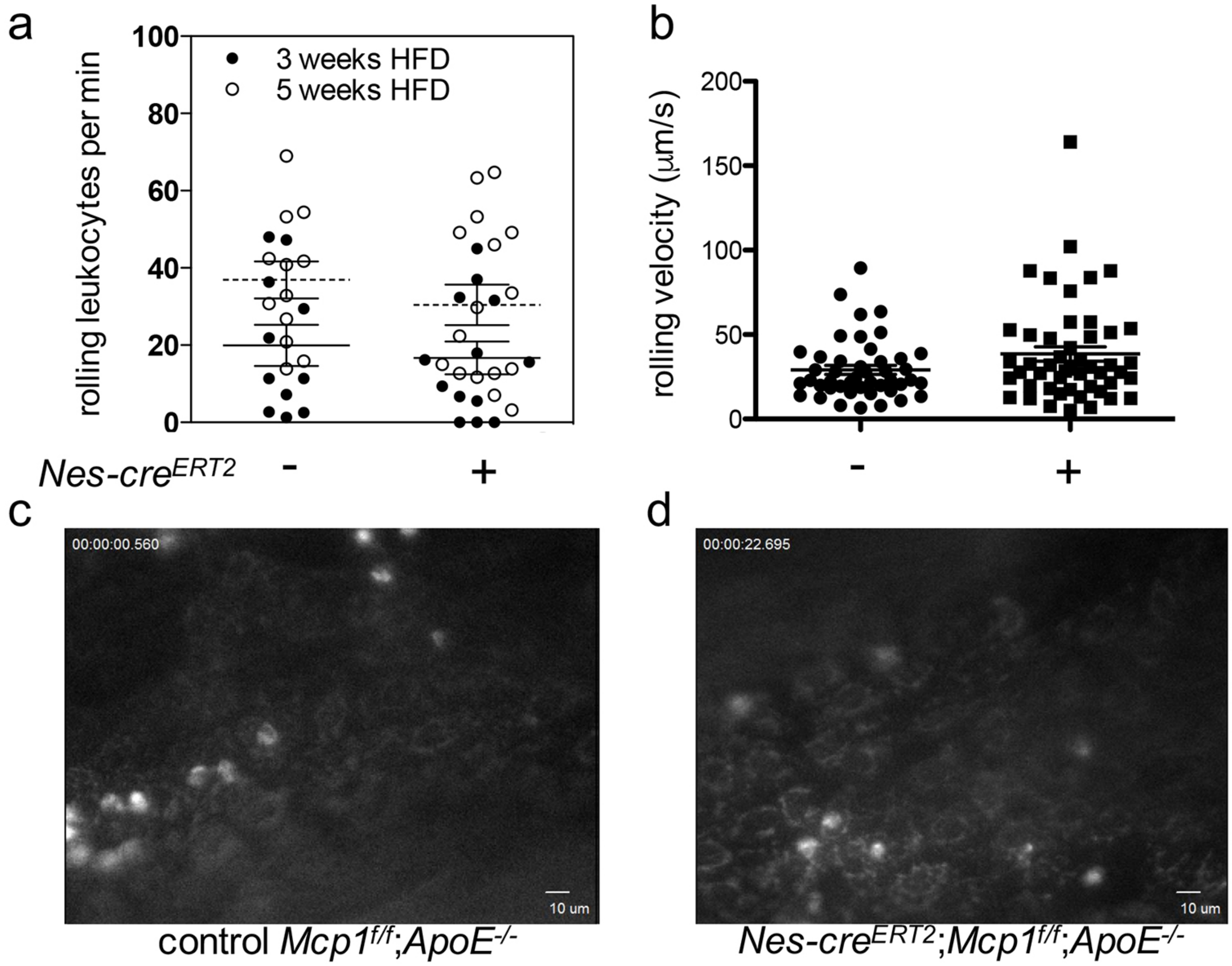

Supplementary Figure 6. Mcp1 deletion in nestin+ cells does not change the velocity or number of rolling leukocytes in the artery wall. (**a**) Number of rolling leukocytes detected by intravital microscopy in the common carotid artery of *Nes-cre*^{ERT2};*Mcp1*$^{f/f}$;*ApoE*$^{-/-}$ mice and *Mcp1*$^{f/f}$;*ApoE*$^{-/-}$ controls fed with HFD for 3 or 5 weeks (n = 6). Data are means ± SEM. (**b**) Rolling velocity. Each dot represents the velocity of one cell (n = 6). (**c**-**d**) High resolution images of rhodamine-labelled leukocytes in the common carotid artery of these mice, near the carotid bifurcation (please see Supplementary Movies 1-2).

Supplementary Figure 7

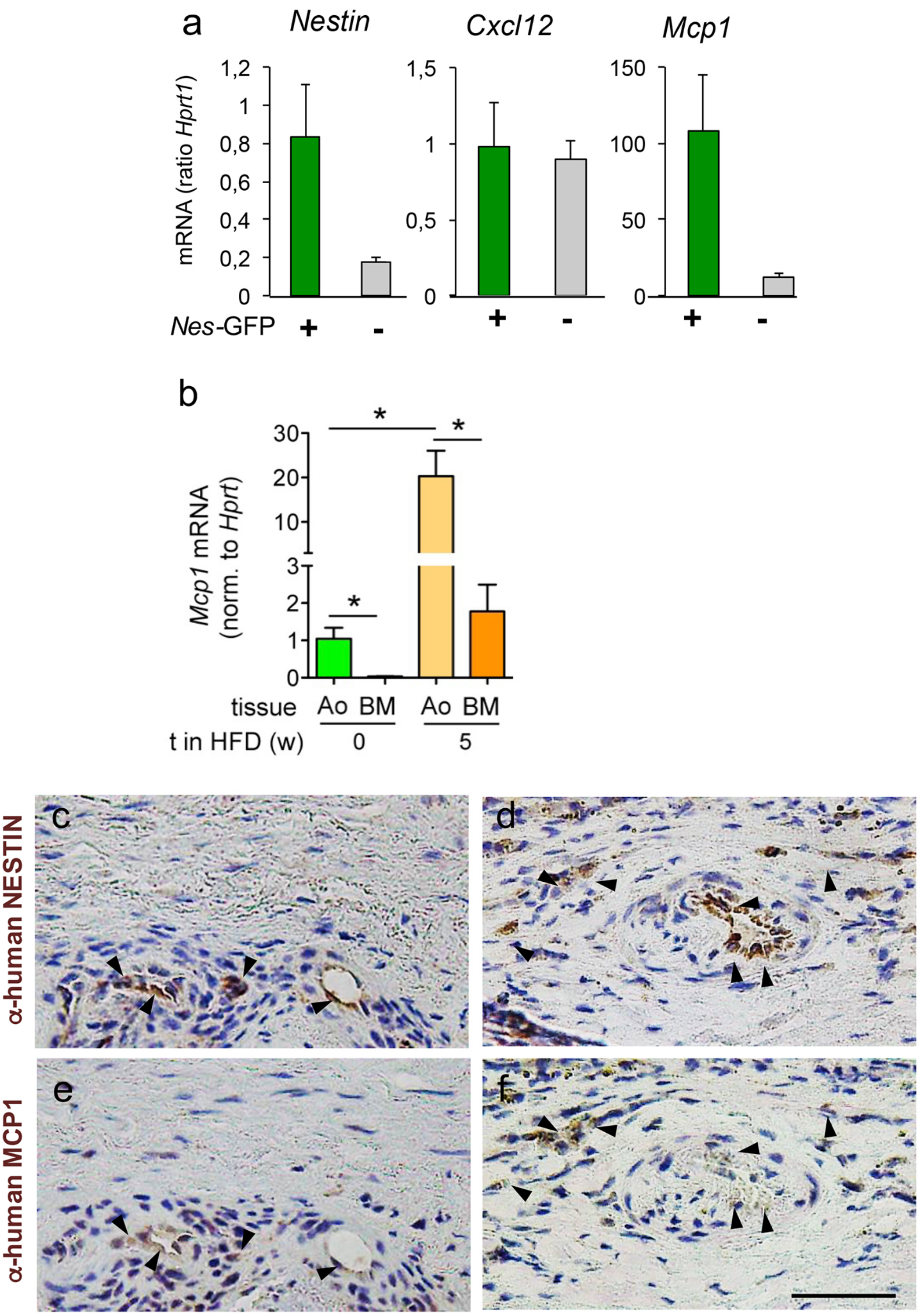

Supplementary Figure 7. Nestin$^+$ cells in the aortic adventitia and media express Mcp1 in mice and humans. (**a**) Expression (QPCR) of *Nestin*, *Cxcl12* and *Mcp1* mRNA in GFP$^{+/-}$ cells sorted from 15 aortas pooled from *Nes-Gfp* mice. Note the enrichment of *Nestin* and *Mcp1* mRNA expression in GFP$^+$ cells. (**b**) QPCR analysis of Mcp1 mRNA in aorta (Ao) and bone marrow (BM) samples from mice fed with chow or HFD for 8 weeks (aorta, n = 11 and 7. BM, n = 7 and 4). (**c-f**) Immunohistochemistry of (**c-d**) NESTIN and (**e-f**) MCP-1 in human carotid artery samples. Black arrowheads indicate cells that express both proteins in consecutive sections. (**a**) Data are means ± SD. (**b**) Data are means ± SEM; * $p < 0.05$, unpaired two-tailed t test. (**f**) Scale bar, 50 μm.

Supplementary Figure 8

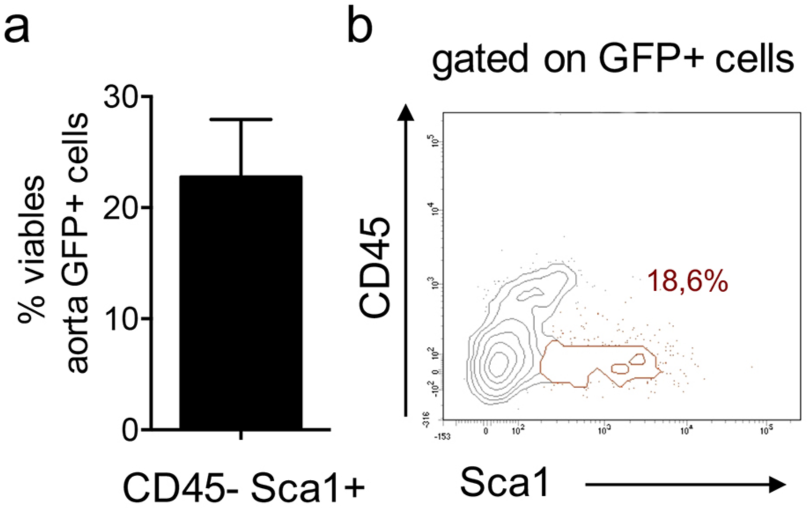

Supplementary Figure 8. Sca1 is expressed by some murine aortic nestin⁺ cells. (**a**) Frequency of aortic GFP+ stromal cells expressing Sca1 marker in *Nes-gfp* mice (*n* = 4). Data are mean ± SEM. (**b**) Representative flow cytometry diagram of aortic cells from *Nes-Gfp* mice showing CD45 and CD31 expression in GFP+ cells.

Supplementary Figure 9

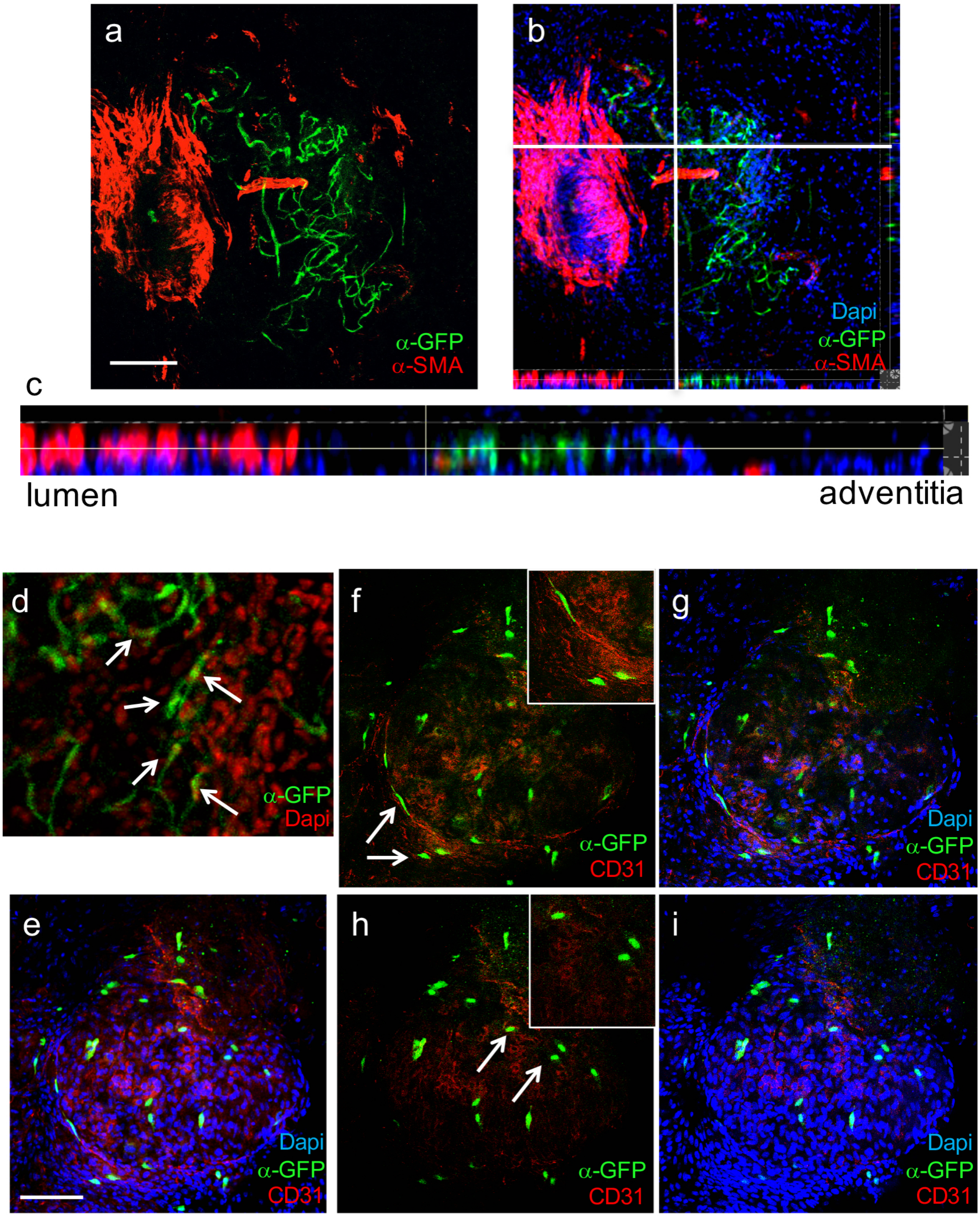

Supplementary Figure 9. Nestin^{+} cells contribute to atheroma plaque formation. (**a**) Whole-mount immunostaining of an atheroma plaque using anti-GFP (far-red channel shown in green pseudocolor) and anti-smooth muscle actin (SMA, red) antibodies. Note that GFP^{+} cells inside the adventitia near to the atheroma plaque do not coincide with SMA^{+} cells. (**b**) Single image corresponding to the middle of the stack (stack 7/14) showing dapi staining. (**c**) Stack orthogonal projection. (**d**) Zoom from stack projection showing nuclear staining (Dapi, red) in cells labeled using anti-GFP antibodies (far-red channel in green). Arrows point toward individual nucleated GFP+ cells. (**e**) Maximal-Z projection of whole-mount immunostaining of atheroma plaque using anti-GFP (far-red channel shown in green pseudocolor) and anti-CD31 (red) antibodies. (**b**, **d**, **g-e**, **i**) Nuclei have been counterstained with dapi (**b**, **g-e**, **i**, blue; **d**, red). (**f-i**) Stack projections showing the presence of (**f-g**) GFP+ CD31+ cells and (**h-i**) GFP+ CD31- cells in the plaque. Insets depict representative examples marked by arrows.

Supplementary Figure 10

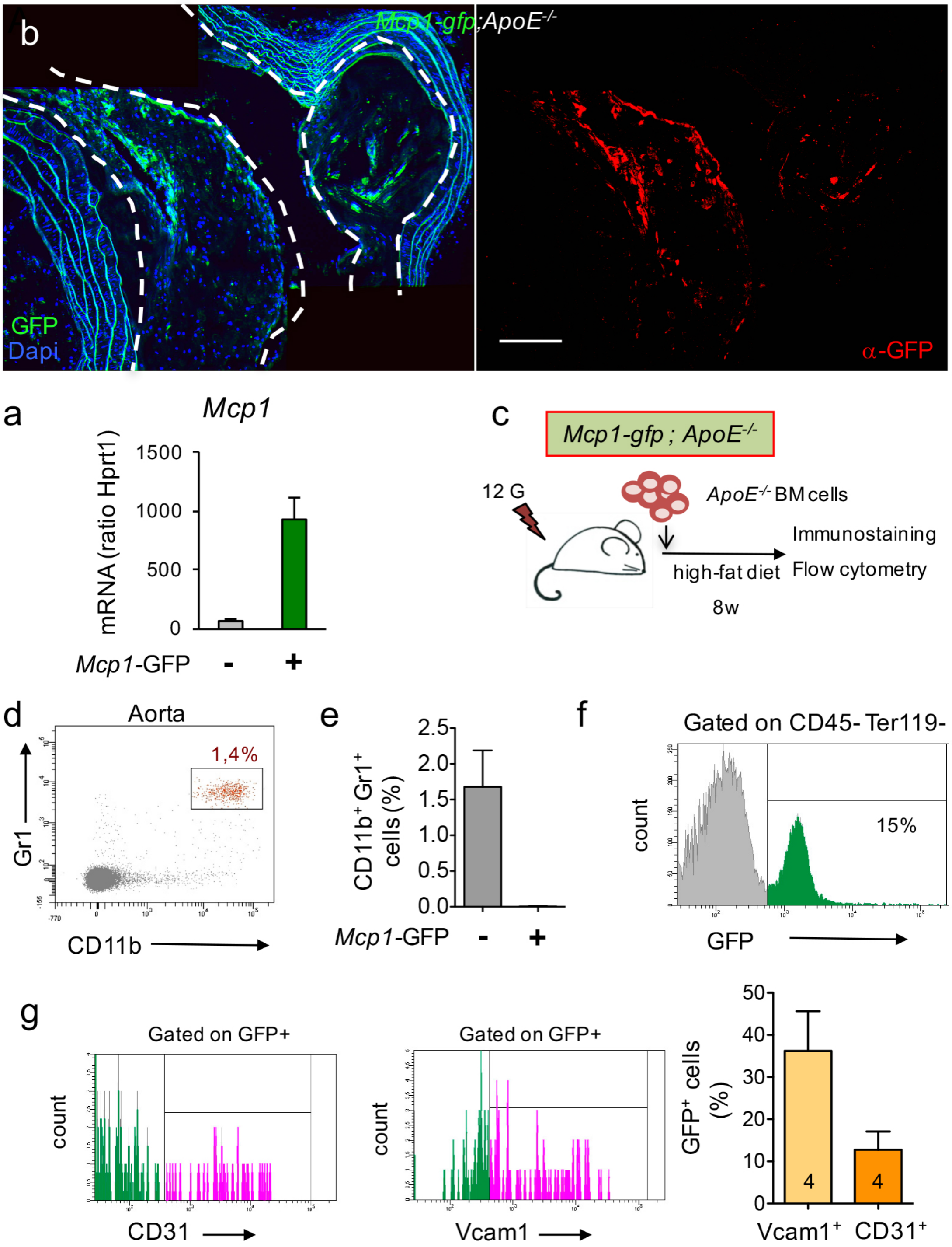

Supplementary Figure 10. Endothelial and mesenchymal cells of the artery wall express Mcp1. (**a**) Expression (QPCR) of *Mcp1* mRNA in GFP$^{+/-}$ cells sorted from 15 pooled aortas of *Mcp1-gfp* mice. Data are means ± SD. (**b**) Representative aortic arch section of *Mcp1-gfp;ApoE*$^{-/-}$ mice fed with HFD for 2 months, immunostained with anti-GFP antibody. Dashed lines delimit the atheroma plaque. Scale bar, 100 μm. (**c**) Experimental design used to detect Mcp1^{+} aortic stromal cells. *Mcp1-gfp;ApoE*$^{-/-}$ mice were lethally irradiated and transplanted (i.v.) with one million *ApoE*$^{-/-}$ bone marrow nucleated cells. Mice were allowed to recover (4 weeks) and were fed with HFD for 8 weeks. (**d-e**) Representative flow cytometry diagram (**d**) and frequency (**e**) of CD11b^{+} Gr1^{+} myeloid cells in the aorta of these mice (n = 6). (**f**) Flow cytometry histogram showing GFP expression in aortic non-hematopoietic cells. The frequency of GFP^{+} cells is indicated. (**g**) Flow cytometry histograms showing the expression of Vcam1 and CD31 in Mcp1-GFP^{+} aortic cells (n = 4). The frequencies are indicated. Data are means ± SEM; n values are indicated inside bars.

Supplementary Figure 11

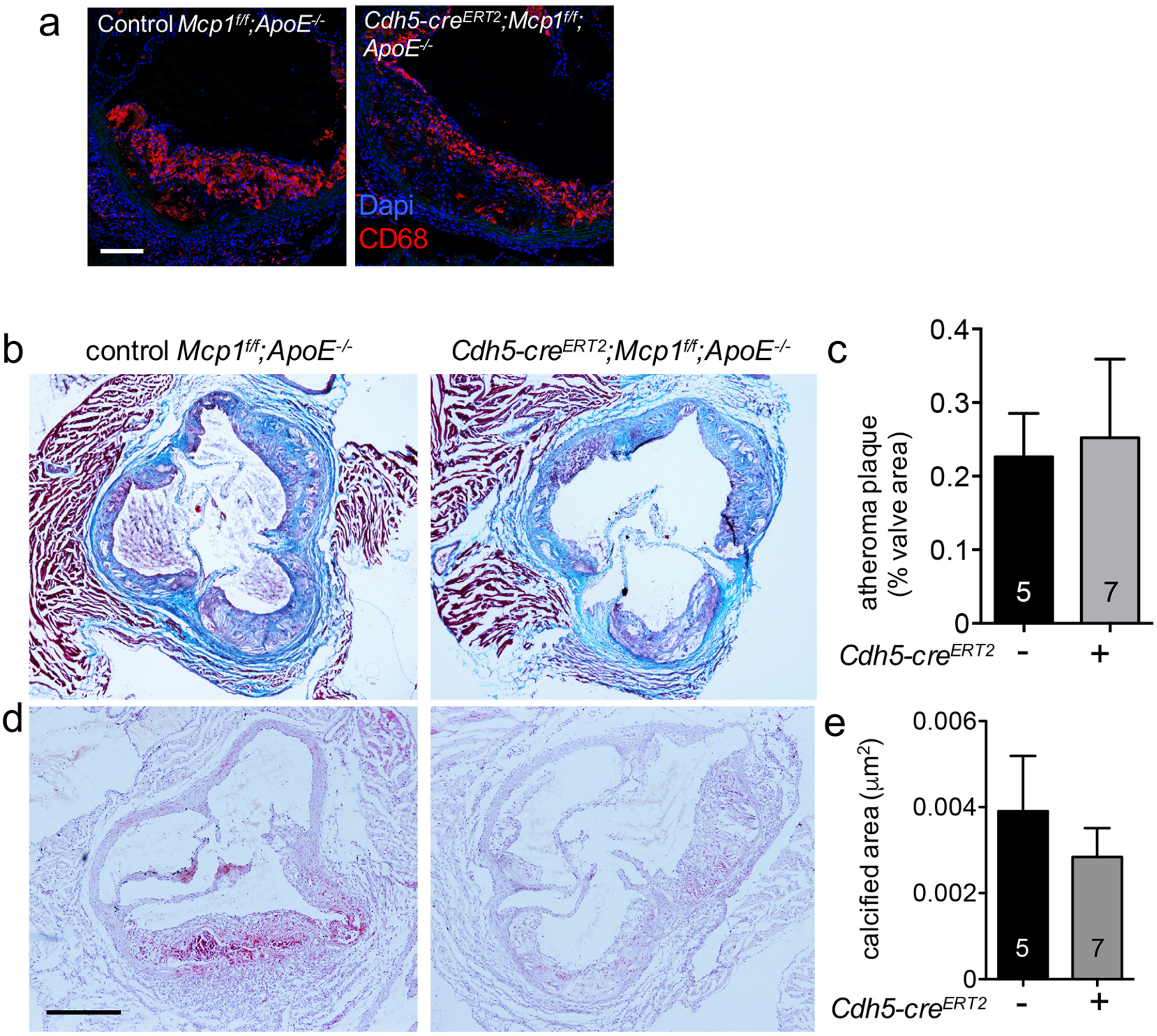

Supplementary Figure 11. Mcp1 deletion in endothelial cells does not change atheroma plaque size or calcification. (**a**) Immunostaining of CD68 (red) to label macrophages infiltrated in the valves of *Cdh5-cre*^{ERT2};*Mcp1*$^{f/f}$;*ApoE*$^{-/-}$ mice and *Mcp1*$^{f/f}$;*ApoE*$^{-/-}$ controls fed with HFD for 8 weeks. Nuclei were counterstained with Dapi (blue). (**b**) Masson trichromic stainings of aortic valve sections from mice fed with HFD for 8 weeks. (**c**) Atheroma plaque area in 2 aortic valve sections from these mice (*n* = 5-7). (**d**) Representative sections of Von Kossa staining of calcium deposits (black) in the aortic valves of *Cdh5-cre*^{ERT2};*Mcp1*$^{f/f}$;*ApoE*$^{-/-}$ mice and *Mcp1*$^{f/f}$;*ApoE*$^{-/-}$ controls fed with HFD for 8 weeks. (**e**) Calcified area in the aortic valves of these mice (*n* = 5-7). Scale bars, (**a**) 100 µm, (**b**, **d**) 200 µm. (**c**, **e**) Data are means ± SEM; *n* values are indicated inside bars.

Supplementary Figure 12

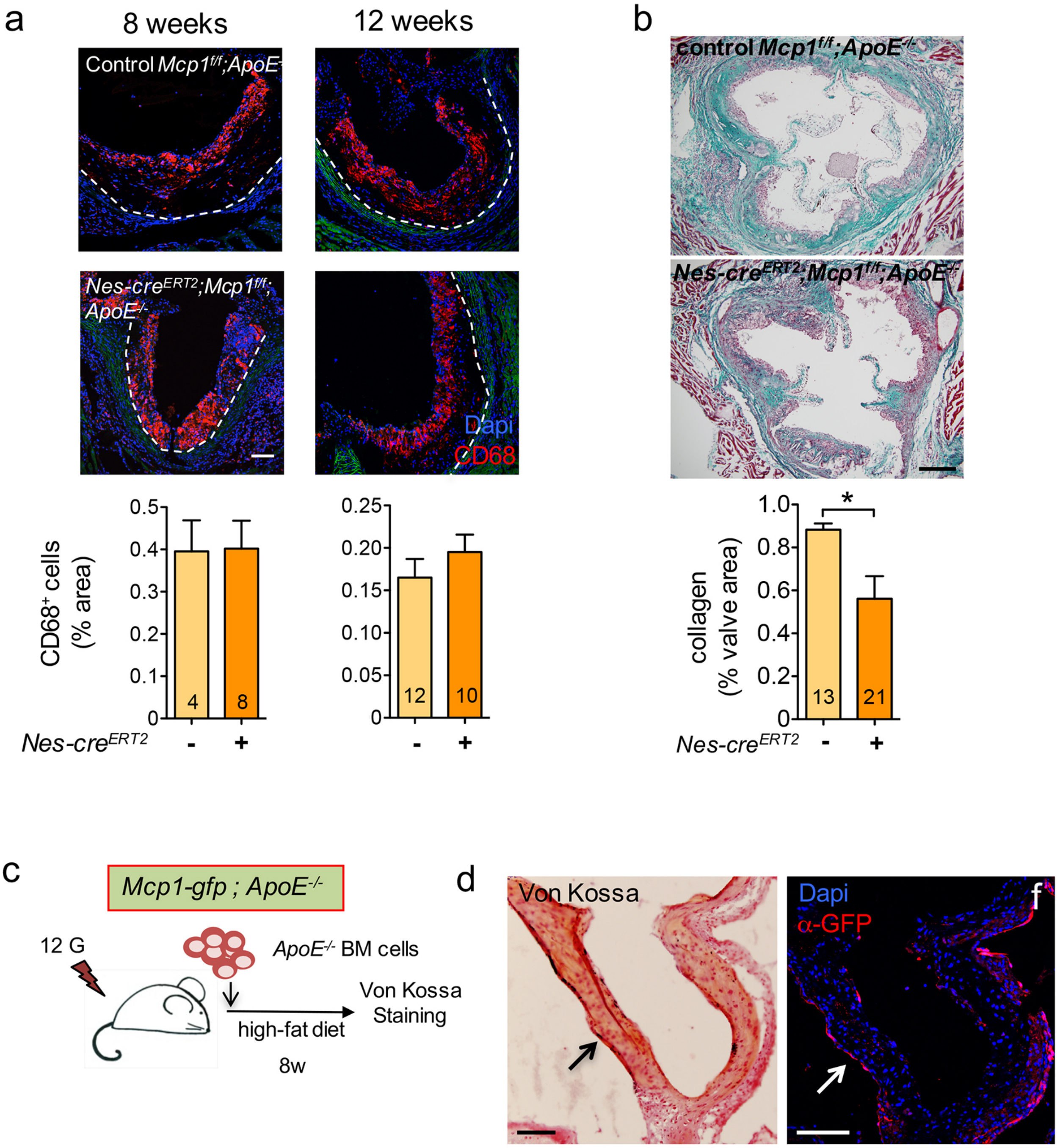

Supplementary Figure 12. Mcp1-producing cells are associated with calcium deposits in the atherosclerotic plaque. (**a**) Immunostaining of CD68 (red) to label macrophages infiltrated in the valves of *Nes-cre*^{ERT2}*;Mcp1*$^{f/f}$*;ApoE*$^{-/-}$ mice and *Mcp1*$^{f/f}$*;ApoE*$^{-/-}$ controls fed with HFD for 8 and 12 weeks. Nuclei were counterstained with Dapi (blue). Bottom bar charts represent the area covered by CD68+ cells in the atheroma plaque of the aortic valves at each time point (n = 4-12). (**b**) Masson trichromic stainings of aortic valve sections from mice fed with HFD for 8 weeks. Green color represents collagen deposition. Bottom bar chart shows the measurement of fibrotic area in 3 aortic valve sections from these mice (n = 13-21). Data are means ± SEM; n values are indicated inside bars. (**c**) Experimental design used to detect Mcp1^{+} aortic stromal cells. *Mcp1-gfp;ApoE*$^{-/-}$ mice were lethally irradiated and transplanted (i.v.) with one million *ApoE*$^{-/-}$ bone marrow nucleated cells. Mice were allowed to recover (4 weeks) and were fed with HFD for 8 weeks. (**d**) Consecutive aortic valve sections from these mice stained with Von Kossa (black) and counterstained with safranin O (left panel), or with anti-GFP antibodies (red) and Dapi (blue) (right panel). The arrow depicts an example of calcified spots near Mcp1^{+} cells (n = 4). Scale bars, (**a**, **d**) 100 μm, (**b**) 200 μm.
